# Algorithm for the Pruning of Synthesis Graphs

**DOI:** 10.1021/acs.jcim.1c01202

**Published:** 2022-04-19

**Authors:** Gergely Zahoránszky-Kőhalmi, Nikita Lysov, Ilia Vorontcov, Jeffrey Wang, Jeyaraman Soundararajan, Dimitrios Metaxotos, Biju Mathew, Rafat Sarosh, Samuel G. Michael, Alexander G. Godfrey

**Affiliations:** National Center for Advancing Translational Sciences, Rockville, Maryland 20850, United States

## Abstract

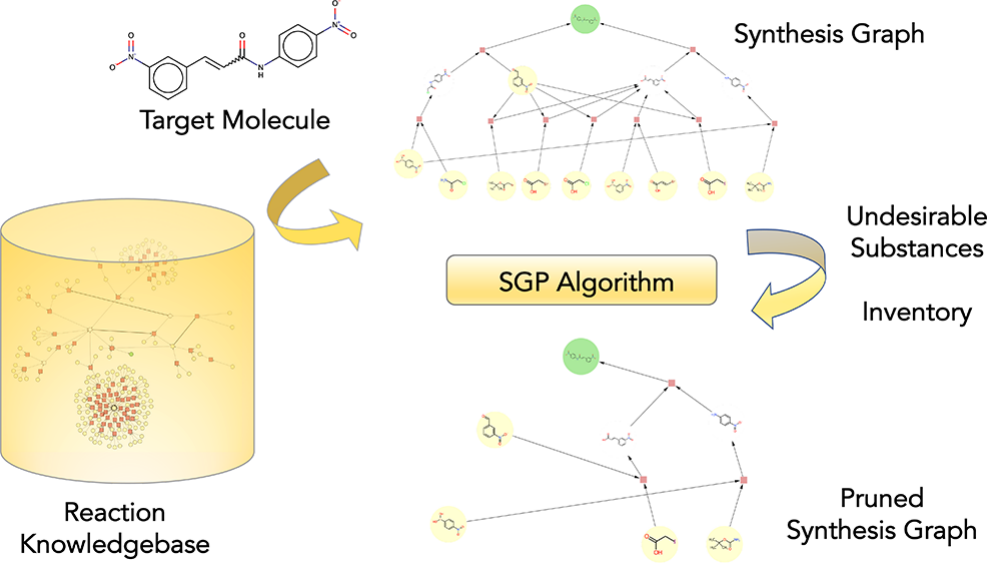

Synthesis route planning is in the core of chemical intelligence that will power the autonomous chemistry platforms. In this task, we rely on algorithms to generate possible synthesis routes with the help of retro- and forward-synthetic approaches. Generated synthesis routes can be merged into a synthesis graph which represents theoretical pathways to the target molecule. However, it is often required to modify a synthesis graph due to typical constraints. These constraints might include “undesirable substances”, e.g., an intermediate that the chemist does not favor or substances that might be toxic. Consequently, we need to prune the synthesis graph by the elimination of such undesirable substances. Synthesis graphs can be represented as directed (not necessarily acyclic) bipartite graphs, and the pruning of such graphs in the light of a set of undesirable substances has been an open question. In this study, we present the Synthesis Graph Pruning (SGP) algorithm that addresses this question. The input to the SGP algorithm is a synthesis graph and a set of undesirable substances. Furthermore, information for substances is provided as metadata regarding their availability from the inventory. The SGP algorithm operates with a simple local rule set, in order to determine which nodes and edges need to be eliminated from the synthesis graph. In this study, we present the SGP algorithm in detail and provide several case studies that demonstrate the operation of the SGP algorithm. We believe that the SGP algorithm will be an essential component of computer aided synthesis planning.

## Introduction

In this study, we describe an algorithm designed to prune a graph that encodes synthesis routes toward a target molecule. A typical scenario where pruning might be necessary is when synthesis routes are extracted from a precomputed reaction knowledge graph. Pruning is induced by eliminating a set of undesirable or unavailable starting materials and/or intermediates from the graph, so that no synthesis route intersects them. This nontrivial problem can be solved using a certain graph representation and associated algorithm operating based on local graph rules. In this study, we describe such an algorithm in detail.

In recent years, chemistry automation^1,2^ has come in the focus of several research and industrial groups interested in the field of drug discovery. In the light of the COVID-19 pandemic, the significance of such a platform cannot be overestimated.^3^ The National Center for Advancing Translational Sciences, National Institutes of Health (NCATS/NIH), has launched “A Specialized Platform for Innovative Research Exploration (ASPIRE)”^4,5^ with the aim of revolutionizing the exploration of the vast (bioactive) chemical space, in order to reduce the translational timeline of delivering novel medical treatments. An autonomous chemistry platform, like ASPIRE, is underpinned by chemistry, informatics, and automation technology. Therefore, it is of outmost importance that we develop novel computational methods which serve as parts of the artificial intelligence (AI) driven chemical intelligence engine of such a platform.

Chemistry automation starts with the selection of one or more target molecule(s) followed by the computer aided synthesis planning (CASP).^6^ Several strategies emerged over the past decades including retrosynthetic analysis,^7−12^ forward synthesis,^13,14^ and reaction prediction.^15−19^ With the help of these methods, it is possible to build a synthesis graph^20,21^ that serves as a map to navigate the synthesis^22^ from starting materials toward the target molecule. Such synthesis graphs can be represented as directed (not necessarily acyclic) bipartite graphs,^23^ constituted by substance and reaction nodes.

A synthesis graph generated for a particular target molecule often includes many potential syntheses out of which only a few are desirable given a set of optimization objectives and constraints.^20,24,25^ Avoiding substances of undesirable property, e.g., toxicity, during the synthesis represents a typical constraint in synthesis planning. Removing substances from the original synthesis graph requires the careful coordination of “pruning” implicated paths while preserving the integrity of paths that provide viable alternative synthesis routes. The algorithm presented in this study is the first to our knowledge that addresses this nontrivial problem.

In the following section, we describe the Synthesis Graph Pruning (SGP) algorithm in detail. The theoretical framework of the SGP algorithm is provided in the “Mathematical Framework of the SGP Algorithm” section in the Supporting Information.

## Computational Methods and Datasets

### Graph Depiction

All graph depiction of this study was created in Cytoscape (v. 3.8.2)^26^ and manually edited in Microsoft Power Point.^27^

### Synthesis Graph Pruning Algorithm

**Figure 1:**
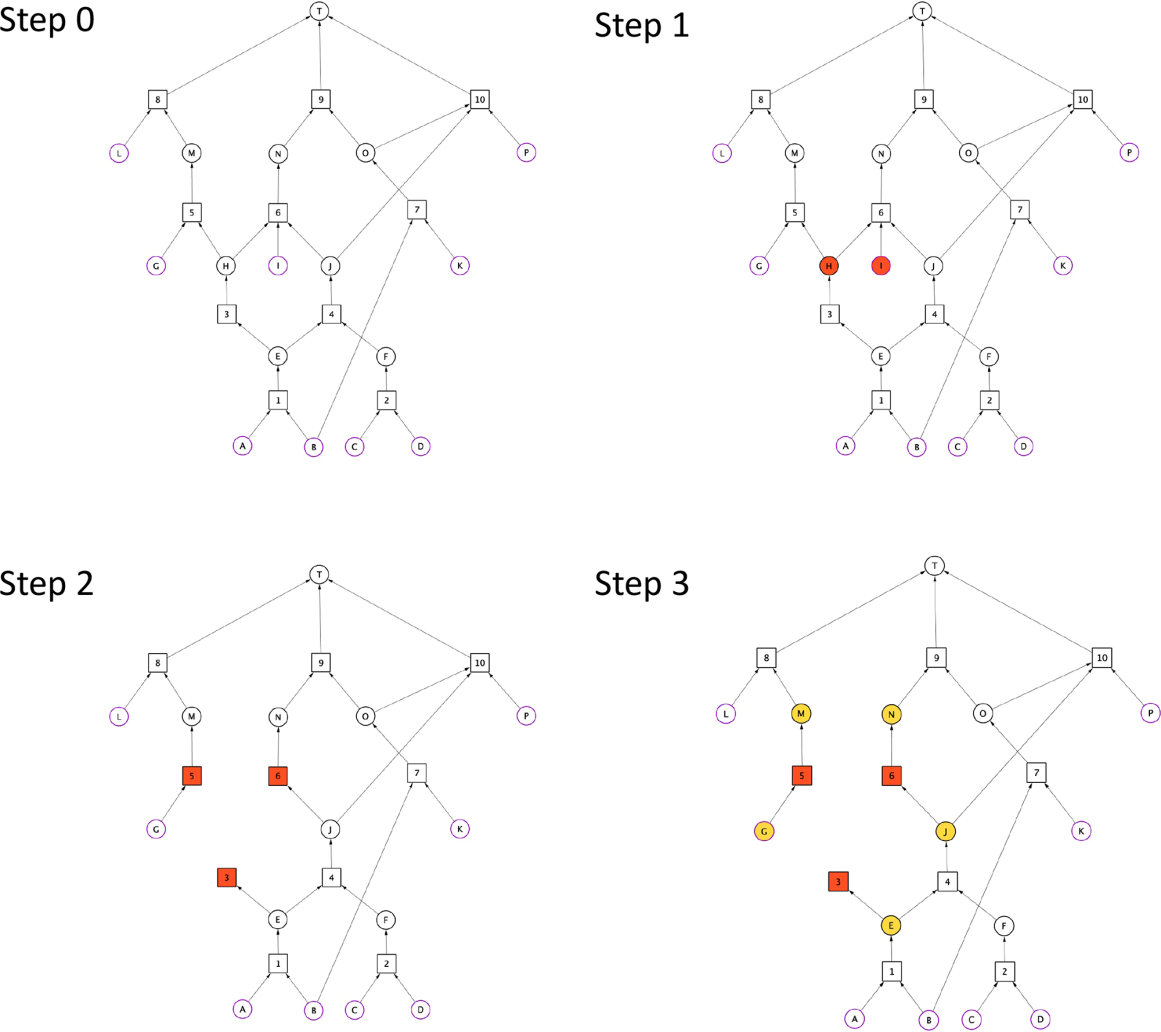
Synthesis Graph Pruning algorithm – part 1. Reaction nodes are represented by squares, whereas substance nodes, by circles. Red nodes are subject to elimination, whereas yellow ones are subject to inspection to be assessed by the elimination criteria (see: Def 9). Substance nodes of purple outline indicate starting materials. The target molecule is denoted by “T”.

**Figure 2:**
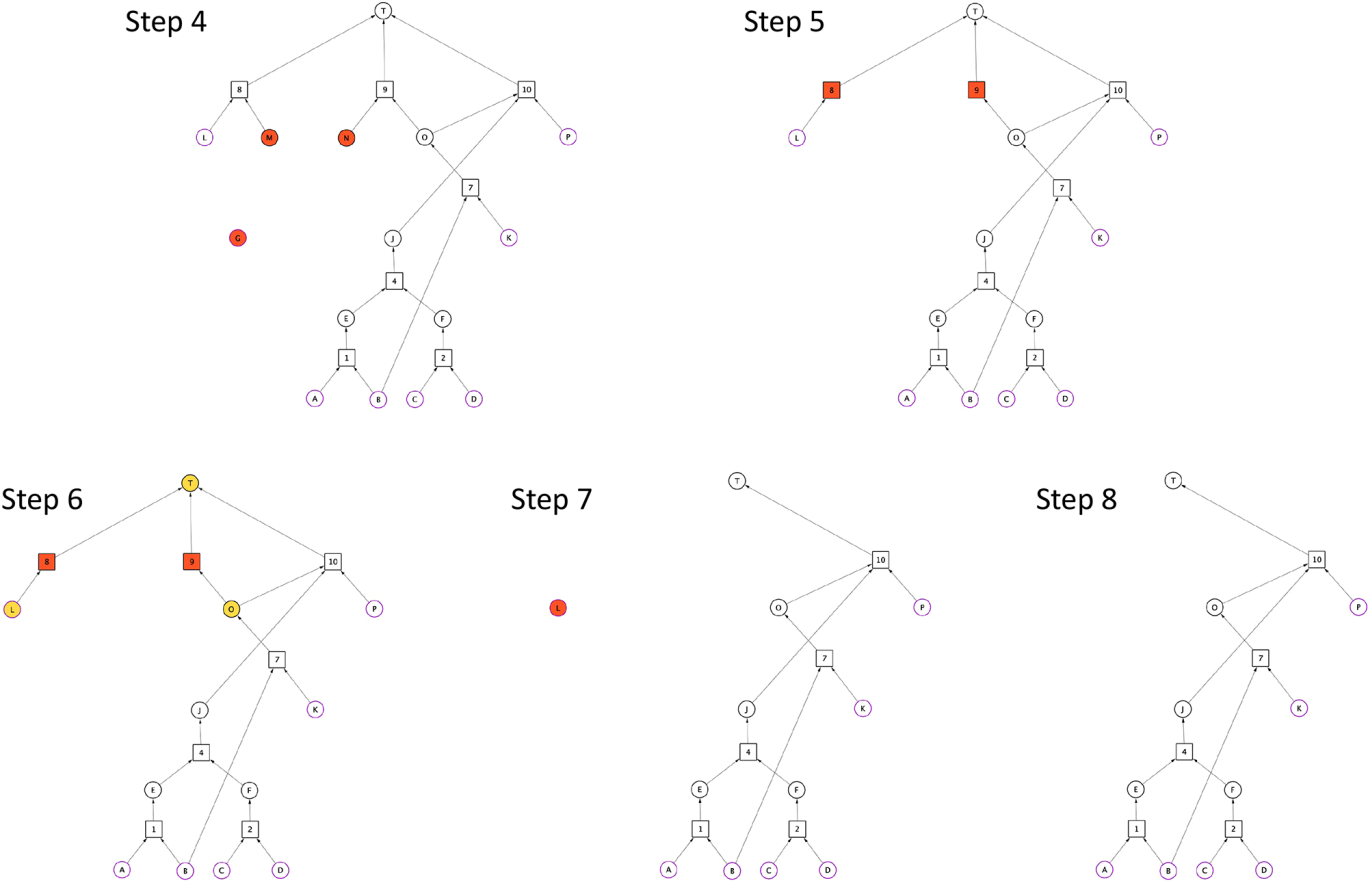
Synthesis Graph Pruning algorithm – part 2. Reaction nodes are represented by squares, whereas substance nodes, by circles. Red nodes are subject to elimination, whereas yellow ones are subject to inspection to be assessed by the elimination criteria (see: Def 9). Substance nodes of purple outline indicate starting materials. The target molecule is denoted by “T”.

In this section, we introduce the SGP algorithm based on Theorem 5 (see the “Mathematical Framework of the SGP Algorithm” section in the Supporting Information). We provide a graphical demonstration of the SGP algorithm in Figures 1 and 2. The prototype of the SGP algorithm was implemented in Python^28^ with the help of the Pandas^29^ and NetworkX^30^ packages.

The SGP algorithm takes a synthesis graph *G* and a set of substance nodes *I* as input. These substance nodes are defined as undesirable for synthesis purposes. The reason why a substance node is undesirable is often linked to toxicity, stability issues, and unavailability in the inventory, to name a few. The SGP algorithm operates iteratively with the help of a rule set that only requires taking the immediate neighborhood of nodes into account to arrive to a decision. Therefore, this rule set can be considered as a local rule set (see Def 9 in the Supporting Information).

First, reaction nodes connected to substances in *I* are marked for deletion, followed by the deletion of nodes in *I*. Next, substance nodes connected to the marked reaction nodes are marked for inspection. Once the reaction nodes are removed, substance nodes marked for inspection are evaluated to see if they need to be removed from *G* in the light of the elimination criteria stated in Theorem 1 and Theorem 2 in the Supporting Information. If any of these substance nodes meets the elimination criteria, it will be marked for deletion, while the rest of the substance nodes (marked for inspection) will become unmarked. This process is repeated until no more nodes are marked for deletion or *G* becomes empty.

The operation of the algorithm is demonstrated through an example shown in Figures 1 and 2. The synthesis graph *G* is shown in Figure 1 as step 0. The set of undesirable substances *I* is defined by substances “H” and “I”; therefore, they are marked for deletion in step 1. In step 2, “H” and “I” are removed, which makes nodes 3, 5, and 6 originally connected to either “H” or “I” undefined (see Def 8 in the Supporting Information). Therefore, 3, 5, and 6 are marked for deletion. In step 3, substance nodes “E”, “G”, “J”, “M”, and “N” connected to any of the reaction nodes 3, 5, and 6 are marked for inspection.

In step 4, reaction nodes 3, 5, and 6 are eliminated. After their elimination, “E”, “G”, “J”, “M”, and “N” substance nodes are inspected to decide if they need to be eliminated. We provide a detailed explanation regarding the outcome of this inspection as follows.

The removed reaction node 3 was a child node of “E”. Therefore, we must assess whether “E” has still at least one child node. After the removal of node 3, “E” is still connected to its child node 4; therefore, “E” becomes unmarked.

Removal of node 5 requires inspecting substance nodes “G” and “M”. Node 5 was the child node of “G”. Considering that “G” is left without a child node, “G” is marked for deletion. Furthermore, node 5 was a parent node of “M”. Considering that “M” is not a starting material and that it is left without a parent node, it is marked for deletion.

Removal of node 6 requires inspecting substance nodes “J” and “N”. Node 6 was the child node of “J”. Considering that “J” is still the parent node of node 10, it becomes unmarked. Furthermore, node 6 was a parent node of “N”. Considering that “N” is not a starting material and that it is left without a parent node, it is marked for deletion.

In step 5, “G”, “M”, and “N” substance nodes are eliminated and reaction nodes 8 and 9 are marked for deletion, as they have become undefined. In step 6, substance nodes “L”, “O”, and “T” are marked for inspection. In step 7, nodes 8 and 9 are eliminated. Following the logic as described in step 4, nodes “O” and “T” become unmarked, whereas “L” is marked for deletion.

In step 8, “L” is the only node that is eliminated. Considering that at this point “L” is no longer connected to any reaction nodes, the iteration process terminates. The graph shown in step 8 is the output of the SGP algorithm, and in this example, it is the only viable (see Def 7 in the Supporting Information) synthesis route *W* leading to target molecule “T” in the synthesis graph *G*.

Note that it may happen that a substance node is connected to multiple reaction nodes. If at least two of these reactions are eliminated in the same iteration step and the substance is the parent node of one of these reactions and the child node of the other one, then the elimination criteria need to be assessed for two scenarios. We need to consider if the substance node has any child nodes left. Additionally, we need to consider if the substance has any parent nodes left and whether the substance is a starting material. If any of the respective elimination criteria become true at the given iteration step, then the substance node will be marked for deletion. Such a scenario is not present in the current example.

Although this example demonstrates a scenario when the SGP algorithm is terminated in eight steps, it can be seen that repeating steps 2–4 will lead to the iterative pruning of any synthesis graph *G*, which will terminate in a deterministic manner.

### USPTO Reaction Dataset

We created a reaction knowledgebase using a 101,903 size subset of the Unites States Patent Office (USPTO) reaction dataset.^31^ This knowledgebase can be used to perform precedent-based synthesis route design.^12^ Synthesis graphs were extracted from this knowledgebase using graph traversal. The method of building the reaction knowledgebase and the graph traversal is out of the scope of this study but will be described in detail in a subsequent manuscript. Nonetheless, all of the synthesis graphs that are the subject of investigation in this study and were extracted from the reaction knowledgebase are available for the reproduction of the analyses (see the “Data and Software Availability” section).

Due to the lack of inventory information in the USPTO reaction dataset, it was necessary to generate this attribute artificially for substances. To this end, reactants of the USPTO subset were identified as starting materials, i.e., available from the inventory, if their molecular weight (MW) is less than 200. The analysis was done in KNIME (v. 4.3.2)^32^ utilizing RDKit nodes.^33,34^ This resulted in considering 17,441 substances as starting materials. Furthermore, all substance nodes with an in-degree of zero in the original (input) synthesis graphs were considered as starting materials.

### SAVI Dataset

In addition to the USPTO database, we integrated a subset of the Synthetically Accessible Virtual Inventory (SAVI) dataset^14,35^ into our reaction knowledgebase. The details of selecting this subset of the SAVI dataset are outside the scope of this study. Nonetheless, our reaction knowledgebase contains 151,306 reactions involving 135,445 substances originating from the SAVI dataset.

## Results and Discussion

### Case Studies

#### Case 1 – Alternative Synthesis Route to a Key Intermediate

**Figure 3:**
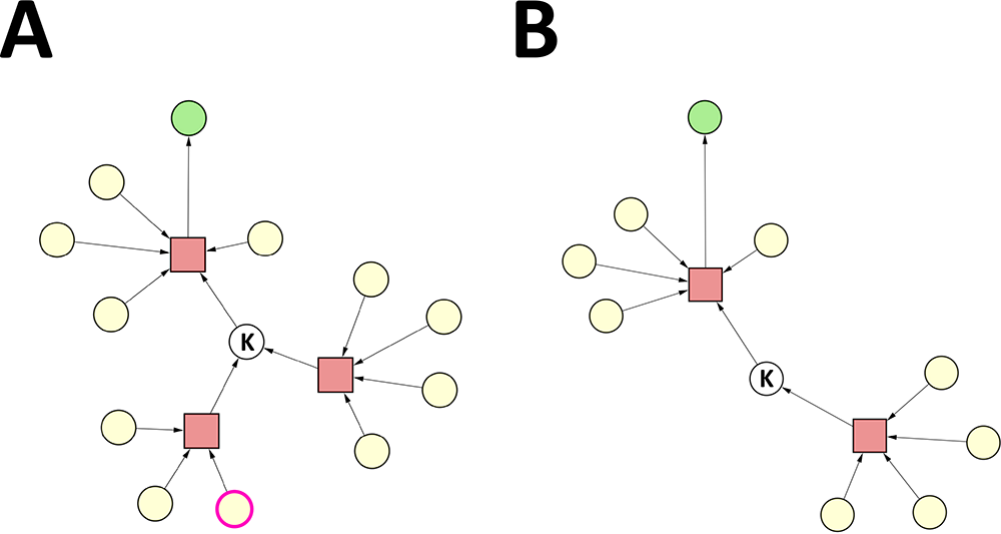
Case 1. Reaction nodes are represented by squares, whereas substance nodes, by circles. Color code of the substance nodes: green, target molecule; yellow, starting material; magenta outline, undesirable substance; white, intermediate. (A) Original synthesis graph. The single undesirable substance is a starting material. (B) Pruned synthesis graph.

The subject of the first case study is a simple synthesis graph which consists of three reactions (see Figure 3A). According to the synthesis graph, there is only one reaction considered as the last step in a multistep synthesis, which produces the target molecule. One of the reactants of the last reaction (yielding the target molecule) is an intermediate which is not a starting material; i.e., it is not readily available from the inventory. Therefore, it needs to be synthesized. The position of its intermediate (labeled as “K”) in the synthesis graph can therefore be considered as a key intermediate. As can be seen, two reactions exist in the synthesis graph to synthesize this key intermediate.

In the synthesis graph, we marked one substance (originally a starting material) as undesirable, which leaves one of those reactions undefined. However, the other reaction (of four reactants/reagents) still leads to the same key intermediate. Running the SGP algorithm on the original synthesis graph leads to the elimination of the reaction node associated with the undesirable substance, as well as the elimination of respective reactant/reagent nodes (see Figure 3B). Note that it is sufficient to mark any of the reactants/reagents of a reaction to make it undefined, and to lead to its elimination. Nonetheless, the SGP algorithm identified that the key intermediate does not need to be eliminated, considering that an alternative synthesis route exists that produces it, i.e., the reaction of four reactants/reagents. As a result, the pruned synthesis graph consists of one viable synthesis route to the target molecule.

#### Case 2 – Multiple Undesirable Substances and Alternative Synthesis Route to a Key Intermediate

**Figure 4:**
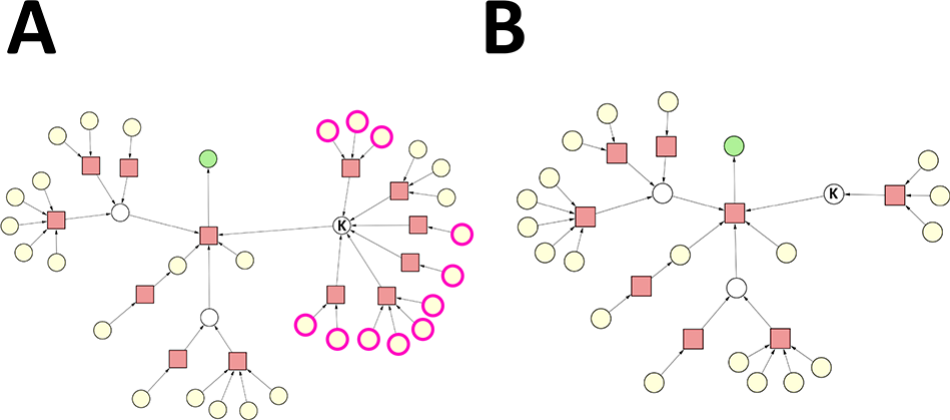
Case 2. Reaction nodes are represented by squares, whereas substance nodes, by circles. Color code of the substance nodes: green, target molecule; yellow, starting material; magenta outline, undesirable substance; white, intermediate. (A) Original synthesis graph. All undesirable substances are starting materials. (B) Pruned synthesis graph.

As compared to case 1, case 2 examines a more complex synthesis graph (see Figure 4A). In this graph, the intermediate (labeled as “K”) associated with six reactions is the center of our investigation. Considering its position in the synthesis graph, it can be deemed as a key intermediate, similarly to the key intermediate of case 1. We marked all of the reactants/reagents as undesirable substances of all but one of those six reactions. On the other hand, none of the reactants/reagents of the remaining one reaction were marked as an undesirable substance. We analyze this scenario in an analogous manner to case 1. That is, all five reactions associated with undesirable substances will be eliminated as a result of applying the SGP algorithm on the synthesis graph, as well as the respective reactants/reagents (see Figure 4B). Since one reaction still exists that leads to a key intermediate, the pruned synthesis graph contains at least one viable synthesis route to the target molecule. In fact, it contains multiple viable synthesis routes, but that is outside of the scope of this case.

#### Case 3 – No Viable Synthesis Route Due to a Key Intermediate That Cannot Be Synthesized

**Figure 5:**
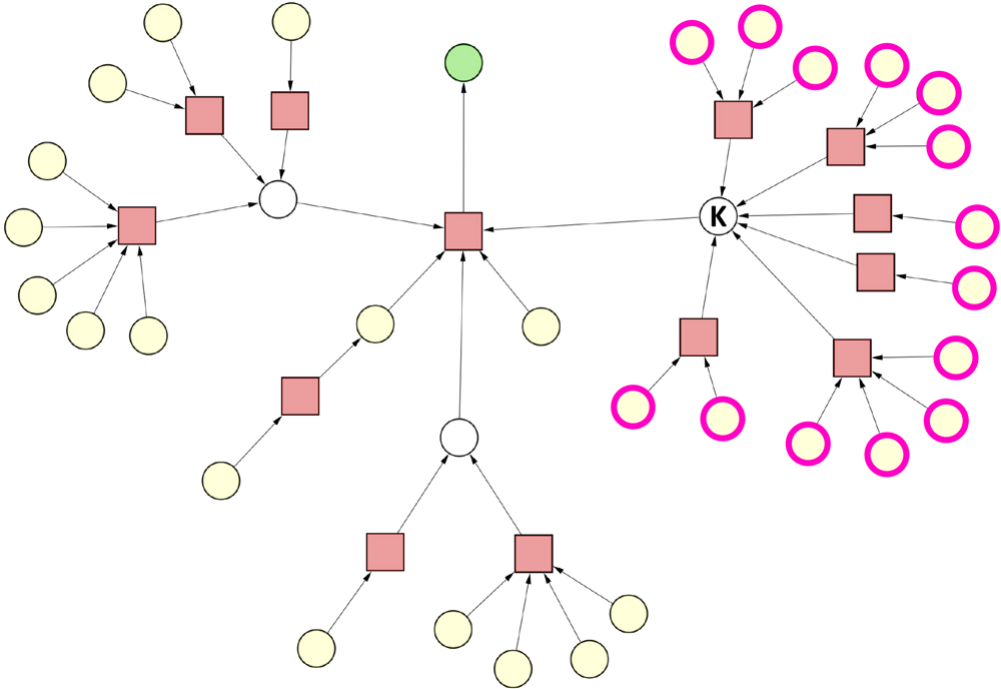
Case 3. Reaction nodes are represented by squares, whereas substance nodes, by circles. Color code of the substance nodes: green, target molecule; yellow, starting material; magenta outline, undesirable substance; white, intermediate. All undesirable substances are starting materials.

Case 3 investigates the same input synthesis graph as case 2 with the sole difference being that the set of undesirable substances was selected in a manner that makes it impossible to synthesize the same key intermediate “K” (see Figure 5). Applying the SGP algorithm on the original synthesis graph yields an empty graph (not shown), since no viable synthesis route leads to the target molecule given the constraints at hand.

#### Case 4 – Multistep Synthesis Involving an Intermediate Which Is Available from the Inventory

**Figure 6:**
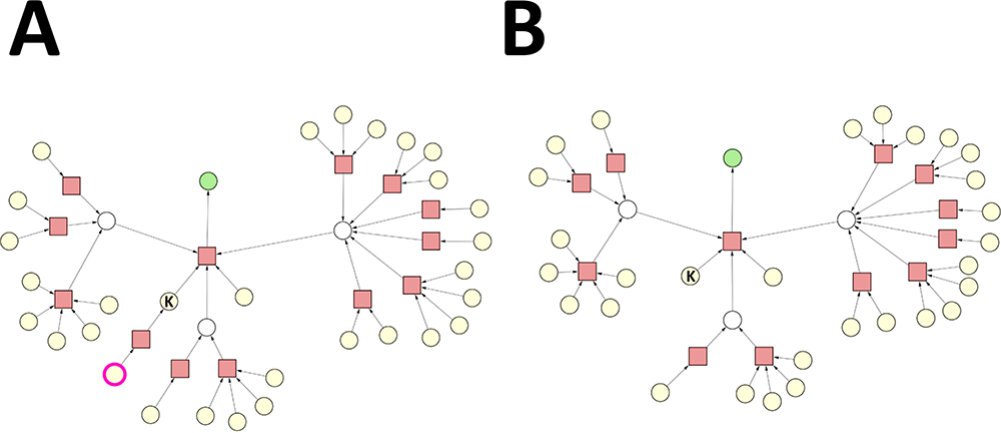
Case 4. Reaction nodes are represented by squares, whereas substance nodes, by circles. Color code of the substance nodes: green, target molecule; yellow, starting material; magenta outline, undesirable substance; white, intermediate. (A) Original synthesis graph. The single undesirable substance is a starting material. (B) Pruned synthesis graph.

Case 4 aims to highlight the importance of distinguishing substances that play an intermediate role in a synthesis graph based on their availability from the inventory. The input synthesis graph of case 4 shown in Figure 6A is nearly identical to that of cases 2 and 3, but it is concerned about another key intermediate (labeled as “K”). Note that substance “K” plays the role of intermediate in this synthesis graph, but it can be considered as a starting material, since it is available from the inventory, as indicated by the color of the node. There is only one substance marked as undesirable in this case, which is the single reactant of the reaction producing substance “K”. Applying the SGP algorithm will eliminate this reaction node from the synthesis graph, and one might expect that substance “K” will also be eliminated accordingly. This is, however, not the case. The SGP algorithm was designed to recognize that “K” is available from the inventory; i.e., it is a readily available starting material. Therefore, the synthesis of the target molecule is still possible, as shown in the pruned synthesis graph (see Figure 6B).

The following question, however, arises: why did the synthesis graph include any subgraphs that converge in the synthesis of substance “K” in the first place? When constructing a synthesis graph, all possible paths are considered up to a certain depth that are relevant for the synthesis of the target molecule at hand. However, some paths in the synthesis graph might also represent a synthesis route to some of the starting materials, i.e., substances available from the inventory.

While this challenge can be addressed programmatically, it is outside of the scope of the SGP algorithm. Nevertheless, a variant of the SGP algorithm could be devised by extending the elimination criteria by an additional one. That is, to also eliminate any reaction nodes, the product of which is a starting material. Of note, this additional criterion will work for single-product reactions, but multiproduct reactions will complicate the landscape. This can be easily seen by considering a scenario when a reaction leads to more than one product, and only one of those products is available from the inventory.

Of note, if substance “K” was not a starting material but an intermediate not available from the inventory, then the application of the SGP algorithm would have led to no viable synthesis route, hence to an empty graph.

#### Case 5 – Removing an Intermediate Destroys All Viable Synthesis Routes

**Figure 7:**
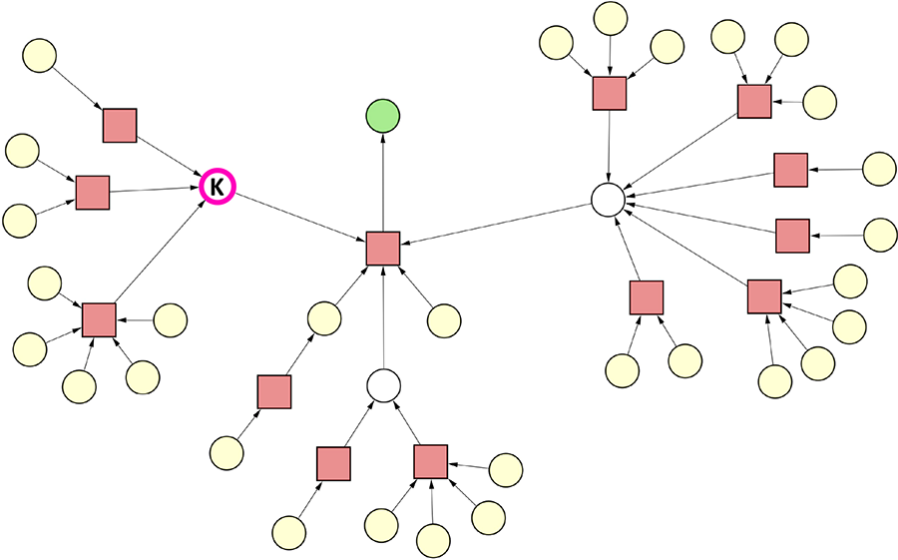
Case 5. Reaction nodes are represented by squares, whereas substance nodes, by circles. Color code of the substance nodes: green, target molecule; yellow, starting material; magenta outline, undesirable substance; white, intermediate. The single undesirable substance “K” is an intermediate.

The input synthesis graph of case 5 shown in Figure 7 is nearly identical to that of case 4; only a different substance (labeled as “K”) was marked as undesirable. Of note, marking “K” as undesirable in this example is hypothetical with the only purpose to demonstrate another aspect of the mechanism of the SGP algorithm. While the input graph of case 5 was chosen to be topologically identical to the input graph of case 4 for the sake of simplicity, the underlying chemistry in a real-life scenario could be completely different. However, due to the topological identity of two graphs, the reactions of the synthesis graph happen to have an identical number of reactants, reagents, and products.

Accordingly, in case 5, substance “K” plays an intermediate role in the synthesis, and it is not available from the inventory. Applying the SGP algorithm in this graph will lead to an empty graph (not shown), since eliminating substance “K” will require the removal the reactions that produce “K” as well as the reaction producing the target molecule. In contrast to case 1, this example demonstrates that an intermediate can also be provided to the SGP algorithm as an undesirable substance. In fact, multiple intermediates can be marked as undesirable substances, as well as a combination of multiple starting materials and intermediates. Examples of these scenarios are provided in cases 8 and 9 (see Figures S8 and S9 in the Supporting Information).

#### Case 6 – Cyclic Paths in a Synthesis Graph

**Figure 8:**
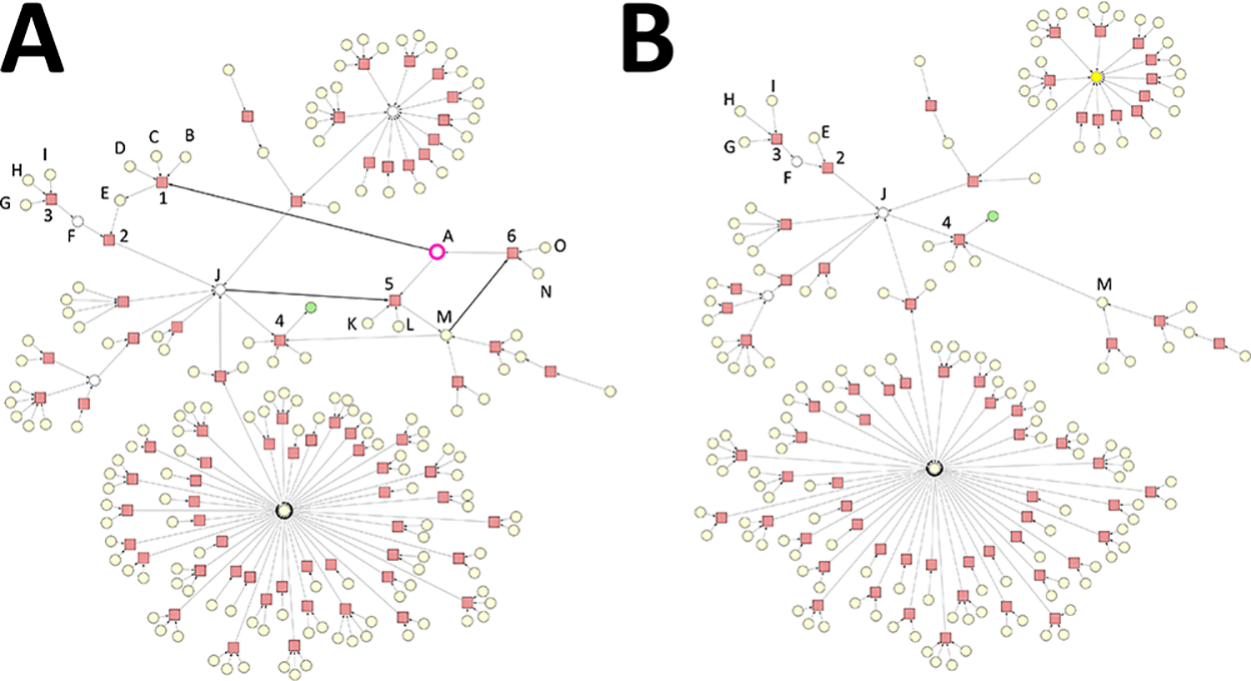
Case 6. Reaction nodes are represented by squares, whereas substance nodes, by circles. Color code of the substance nodes: green, target molecule; yellow, starting material; magenta outline, undesirable substance; white, intermediate. Bold lines indicate artificial relationships that were manually added to a synthesis graph in order to demonstrate a use case. Letters indicate substances, whereas numbers indicate reactions. (A) Original synthesis graph. The single undesirable substance “A” is an intermediate. (B) Pruned synthesis graph.

The previous cases were demonstrating the operation of the SGP algorithm on relatively simple synthesis graphs. Notably, none of those graphs contain a cyclic path. For the sake of demonstrating that the SGP algorithm can handle graphs that include cycles, we created such a graph by manually introducing some artificial edges into a more complex synthesis graph (see Figure 8A). Although this graph was created manually, such synthesis graphs can emerge in a real-life setting.

The synthesis graph on Figure 8A contains two directed cyclic paths that can be defined by the sequence of nodes as follows: “A-1-E-2-J-5-M-6-A” and “A-5-M-6-A”. Substance “A”, an intermediate, was marked as the single undesirable substance.

The results of applying the SGP algorithm on this synthesis graph are shown in Figure 8B. Considering that “A” is an undesirable substance, it is eliminated from the graph. In consequence, reactions “1”, “5”, and “6” become undefined, leading to their elimination from the synthesis graph. This leaves substances “B”, “C”, “D”, and “K”, “L”, and “O” and “N” without a child reaction node. Considering the elimination criterion (see Def 9), that is the out-degree^36^ of these substance nodes being zero, these substance nodes will be eliminated. Substance “E” on the other hand does not need to be eliminated. Although its in-degree^36^ becomes zero, it is a starting material, i.e., readily available from the inventory. The rest of the graph remains unchanged. It can be seen how the SGP algorithm correctly eliminated certain paths from the original synthesis graphs while leaving the viable synthesis routes intact.

#### Case 7 – Intermediate of an Alternative Synthesis Route in a Cyclic Path

**Figure 9:**
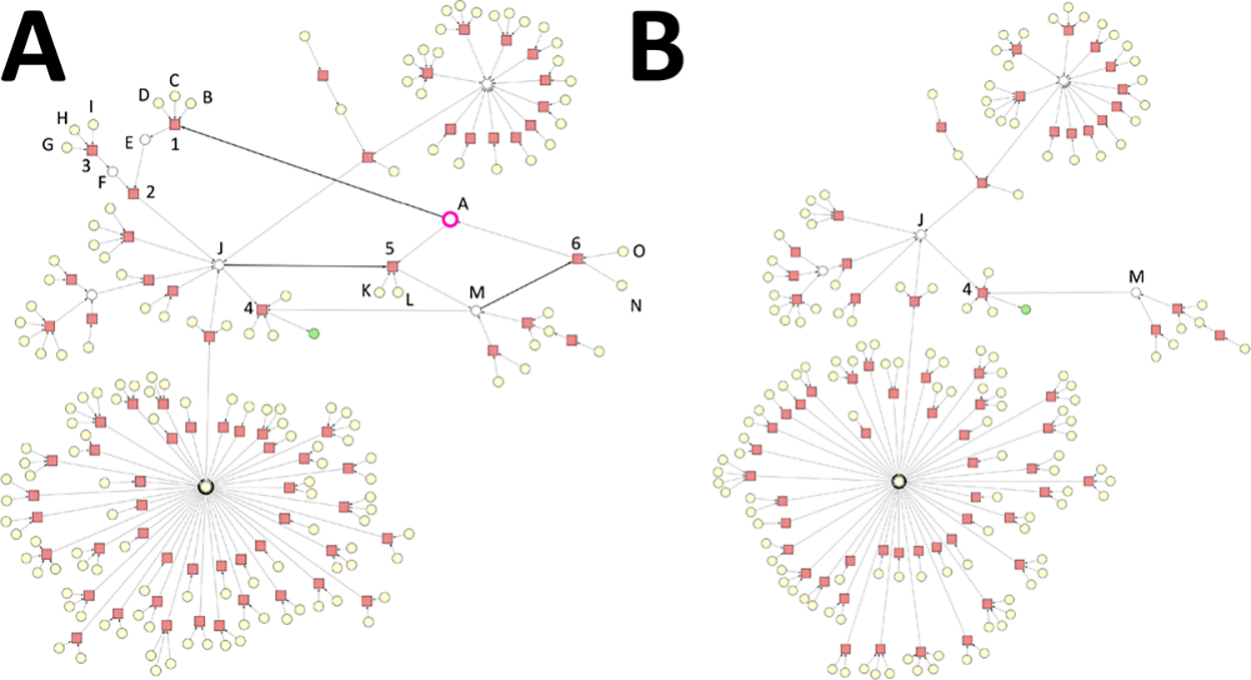
Case 7. Reaction nodes are represented by squares, whereas substance nodes, by circles. Color code of substance nodes: green, target molecule; yellow, starting material; magenta outline, undesirable substance; white, intermediate. Bold lines indicate artificial relationships that were manually added to a synthesis graph in order to demonstrate a use case. Letters indicate substances, whereas numbers indicate reactions. (A) Original synthesis graph. The single undesirable substance “A” is an intermediate. (B) Pruned synthesis graph.

In case 7, we investigate an input graph (see Figure 9A) that is nearly identical to that in case 6. The sole difference is that this time substance “E” is an intermediate unlike in case 6, where it was a starting material. Given the same undesirable substance “A”, SGP leads to the graph shown in Figure 9B. In the resultant graph, all of the nodes that were eliminated in case 6 are also eliminated in case 7. However, in contrast to case 6, the elimination of reaction “1” leads to the elimination of substance “E” considering the elimination criterion (see Def 9); that is, “E” is a substance of in-degree zero and not a starting material. Consequently, reaction “2” becomes undefined which will lead to the elimination of substances “F”, “G”, “H”, and “I” and reaction “3”. Note, labels of nodes were preserved across cases 6 and 7.

### Use Cases Involving Specific Reactions

**Figure 10:**
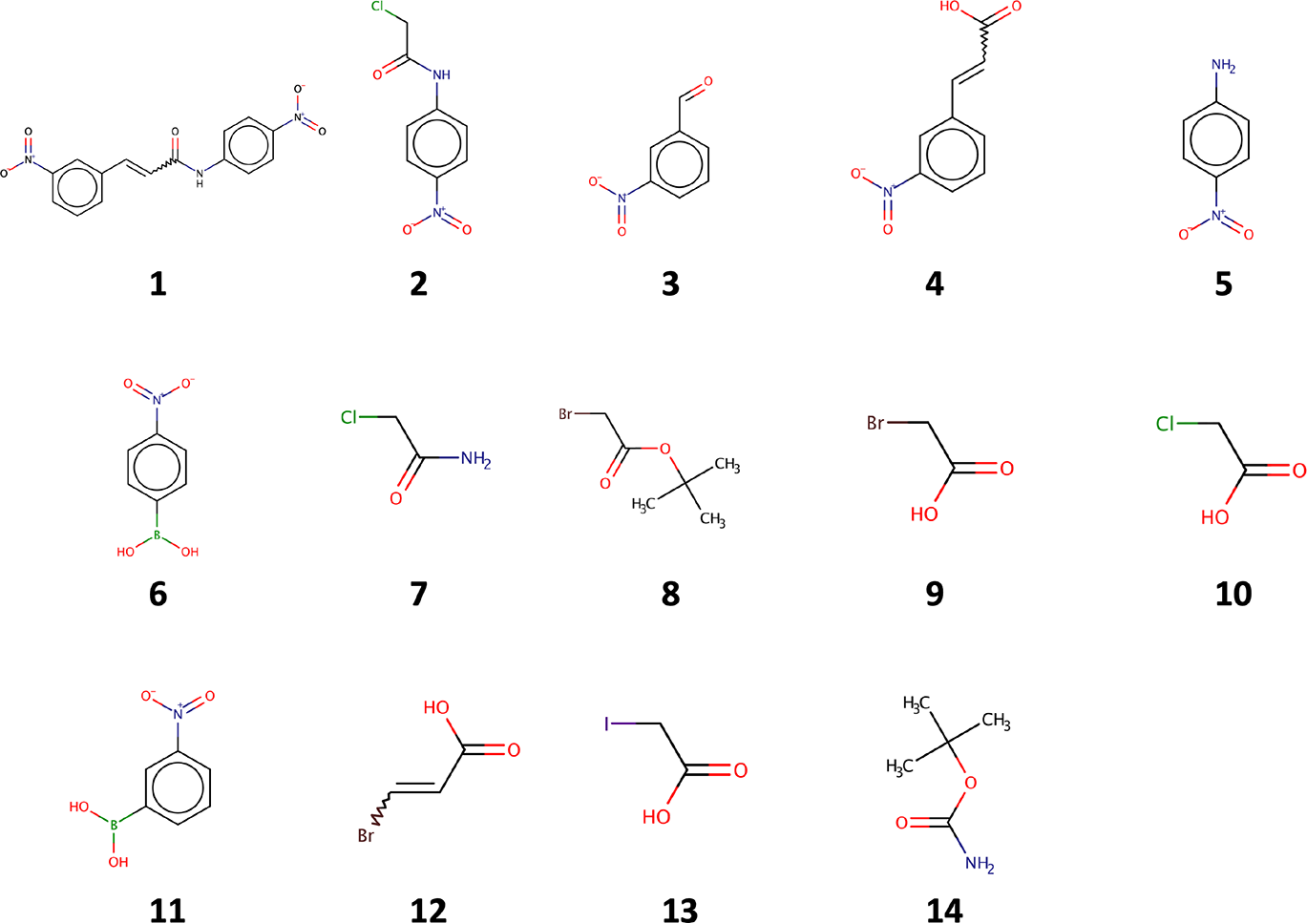
Substances involved in the use cases. The numbering of the substances is in correspondence with the numbering of the substance nodes of the synthesis graphs throughout the case studies. Molecules were depicted with MarvinSketch (v16.12.12) from ChemAxon.^37^

The synthesis graphs involved in the above case studies were created with the purpose of demonstrating the operation of the SGP algorithm. We provide use case examples that are based on specific reactions that were extracted from our reaction knowledgebase. The knowledgebase contains reactions from the USPTO and SAVI databases.^14,31,35^ We extracted one synthesis graph which is used as the underlying graph in all case studies for the sake of simplicity. Besides the underlying synthesis graph, the SGP algorithm takes as input the set of undesirable substances and the set of substances available from the inventory (starting materials). Providing these sets to the algorithm as input is the responsibility of the investigator, as these sets are context dependent. Nevertheless, for demonstration purposes, we make an assumption throughout the use cases as to which substances are considered undesirable and which ones are available from the inventory. While the set of undesirable substances may be overlapping across the various use cases, they need to be considered as independently defined in each use case. The same consideration is true for the set of inventory substances. Substances involved in the use cases are shown in Figure 10, and further details of them are provided in Table S1 in the Supporting Information.

#### Use Case 1

**Figure 11:**
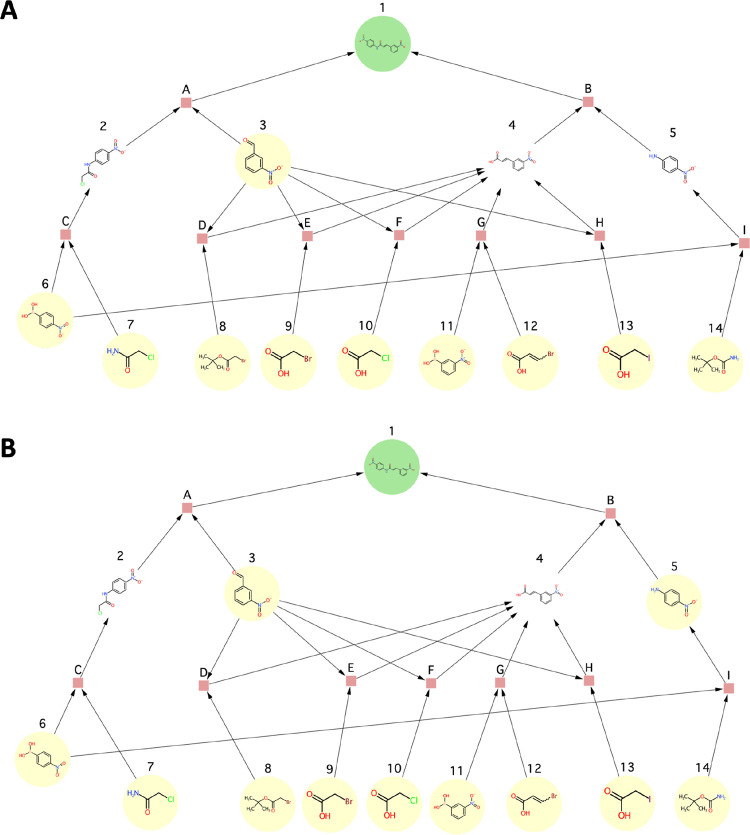
Synthesis graphs involved in the use cases. The numbering of the substance nodes is in correspondence with the numbering of substances shown in Figure 10. Letters denote reaction nodes. Color code of the substance nodes: green, target molecule; yellow, starting material; white (no background color), intermediate. Reactants of a reaction are indicated by edges that start from the substance nodes and end in the reaction node. Products of the reactions are represented by edges that start from the reaction nodes and end in the substance nodes. Molecules were depicted by the “chemViz2” plugin^38^ for Cytoscape. (A) This synthesis graph is the underlying graph in all specific examples except for example 3b. (B) This is the underlying graph of example 3b. Note, the sole difference between the two graphs is whether substance **5** is a starting material or an intermediate.

**Figure 12:**
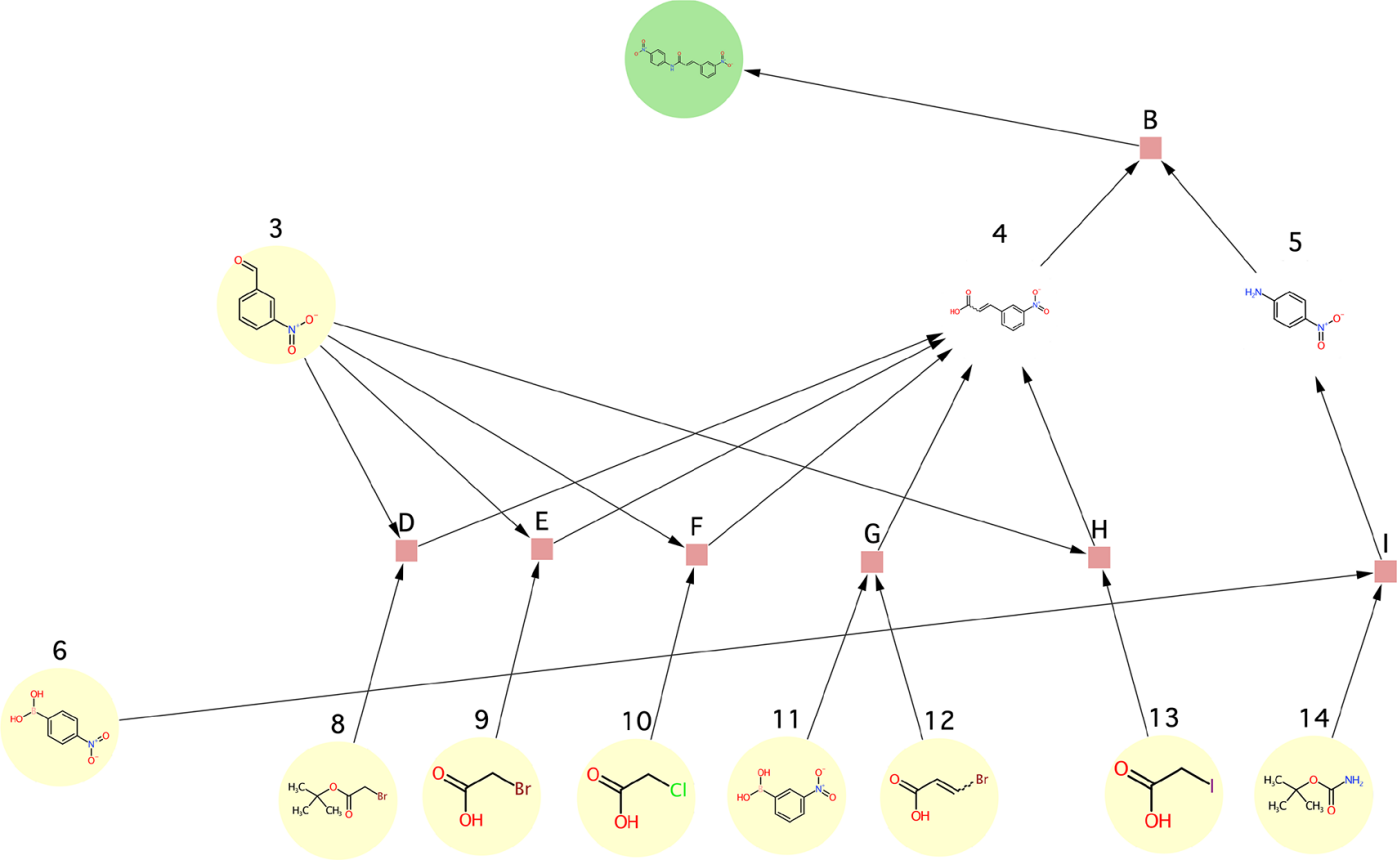
Use case 1 - synthesis graph pruned by the SGP algorithm.

The input synthesis graph to use case 1 is shown in Figure 11A. The undesirable substance was arbitrarily assumed to be substance **2**. Running the SGP algorithm gave rise to the graph shown in Figure 12. Since **2** is an undesirable substance in this example, the SGP algorithm eliminated it from the input synthesis graph. In consequence, reactions A and C become undefined and thus were eliminated as well from the graph. Since substance **7** was no longer connected to any reactions, it is removed from the graph. However, the SGP algorithm correctly recognized that substances **3** and **6** are part of multiple viable synthesis routes and therefore they were not eliminated from the graph. Indeed, substance **6** is the only option in the pruned synthesis graph to synthesize **5** which is essential for reaction B. Keeping **3** in the graph is justified, as it is the source of multiple alternative synthesis routes toward **4**. In the next example, we take a closer look at substance **3**.

#### Use Case 2

As we have seen in the “Use Case 1” section, substance **3** is involved in multiple synthesis routes that include substance **4**. Therefore, we sought to investigate what happens if only substance **3** is assumed to be (arbitrarily) an undesirable substance. The underlying input graph to this use case is identical to that of use case 1 (see Figure 11A).

**Figure 13:**
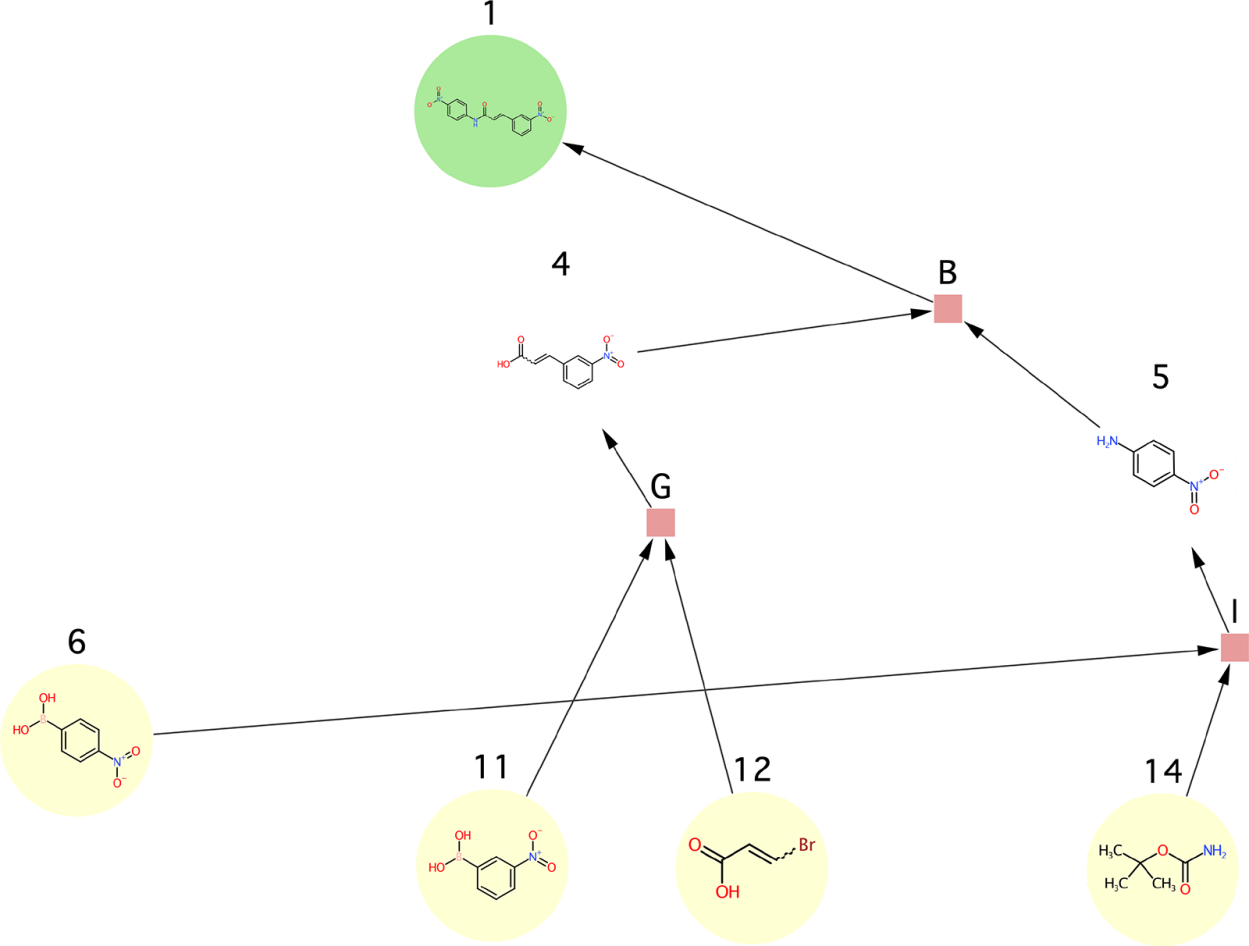
Use case 2 - synthesis graph pruned by the SGP algorithm.

Elimination of **3** as an undesirable substance leads to the elimination of reaction A, substance **2**, and in turn reaction C and substance **7**. Substance **6** is retained in the pruned graph, as it is a reactant to reaction I which is not affected by the elimination of **3**. The reason for this is that a synthesis route exists toward **4** which is independent from **3**. That is, **4** can be synthesized by reaction G involving **11** and **12**. In fact, in the pruned graph, there remains only one viable synthesis route toward **1** as a consequence of the elimination of **3** from the original synthesis graph. The SGP algorithm correctly identified the only viable synthesis route given its input. The pruned graph is shown in Figure 13.

#### Use Case 3

In this use case, we consider two slightly different scenarios, namely, use cases 3a and 3b. Substance **6** was arbitrarily assumed to be the only undesirable one in the graph in both scenarios. While use case 3a takes as input the same input graph that was involved in previous examples (see Figure 11A), use case 3b takes a slightly different input graph as input (see Figure 11B). Note that the sole difference between these input graphs concerns the assumed availability of substance **5** from the inventory. Substance **5** is assumed not to be available from the inventory in the graph shown in Figure 11A in contrast to the graph shown in Figure 11B. This difference will have important implications when pruning the input graph, as we detail below.

##### Use Case 3a

As described above, the input graph of use case 3a is shown in Figure 11A and the undesirable substance was arbitrarily assumed to be **6**. Applying the SGP algorithm on this synthesis graph results in an empty graph (not shown). That is, no viable synthesis route is found toward **1** considering the availability of substances from the inventory and **6** being assumed an undesirable substance. Since **6** cannot be used in the synthesis in this use case, it makes the synthesis of both **2** and **5** impossible. In consequence, **2** and **5** will be eliminated from the synthesis graph, leaving the only two reactions toward **1**, namely, A and B, undefined. Therefore, in the light of the input graph and constraints, the SGP algorithm correctly identifies that no viable synthesis route exists toward **1**.

##### Use Case 3b

**Figure 14:**
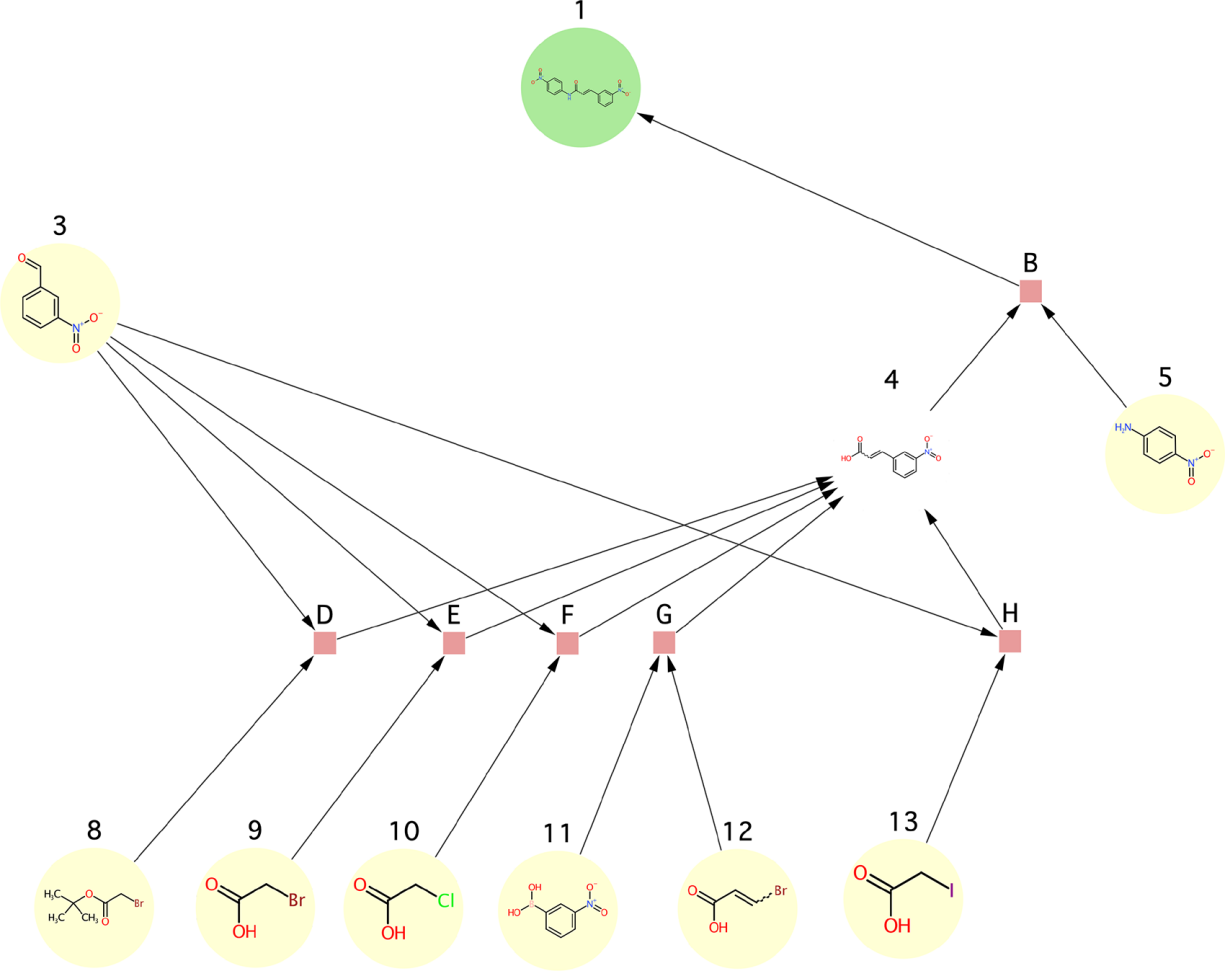
Use case 3b - synthesis graph pruned by the SGP algorithm. Note that the input graph to this use case was the one shown in Figure 11B unlike in the case of all other use cases where the graph shown in Figure 11A was used as the input graph.

Substance **5** was assumed not to be available from the inventory in use case 3a, which made it an “intermediate” according to the terminology of the SGP algorithm. In the current use case, however, substance **5** is assumed to be available from the inventory which makes it a “starting material” in use case 3b. Everything else is identical to use case 3a in terms of the input graph, the undesirable substance (**6**), and the inventory availability of the other substances besides **5**. Applying the SGP algorithm to this synthesis graph results in the pruned graph shown in Figure 14.

Elimination of substance **6** in use case 3b does not lead to the elimination of reaction B. The main reason is that in this use case substance **5** was assumed to be available from the inventory. Therefore, **5** can be used in reaction B despite the elimination of **6** and reaction I. Indeed, **1** can be synthesized starting from **11**, **12**, and **5** via reactions G and B.

As compared to use case 3a, interestingly, **3** is also retained in the pruned graph in use case 3b, providing four more alternative routes involving reactions D, E, F, and H. This is related to the fact that reaction B remained a possibility despite the elimination of **6** and reactions A and I, in contrast to use case 3a. The difference can be solely accounted for by the assumed availability of **5** from the inventory in use case 3b as opposed to use case 3a. That is why taking into account the availability from the inventory is a key concept in the SGP terminology and a key property in the mechanism of the algorithm.

#### Use Case 4

**Figure 15:**
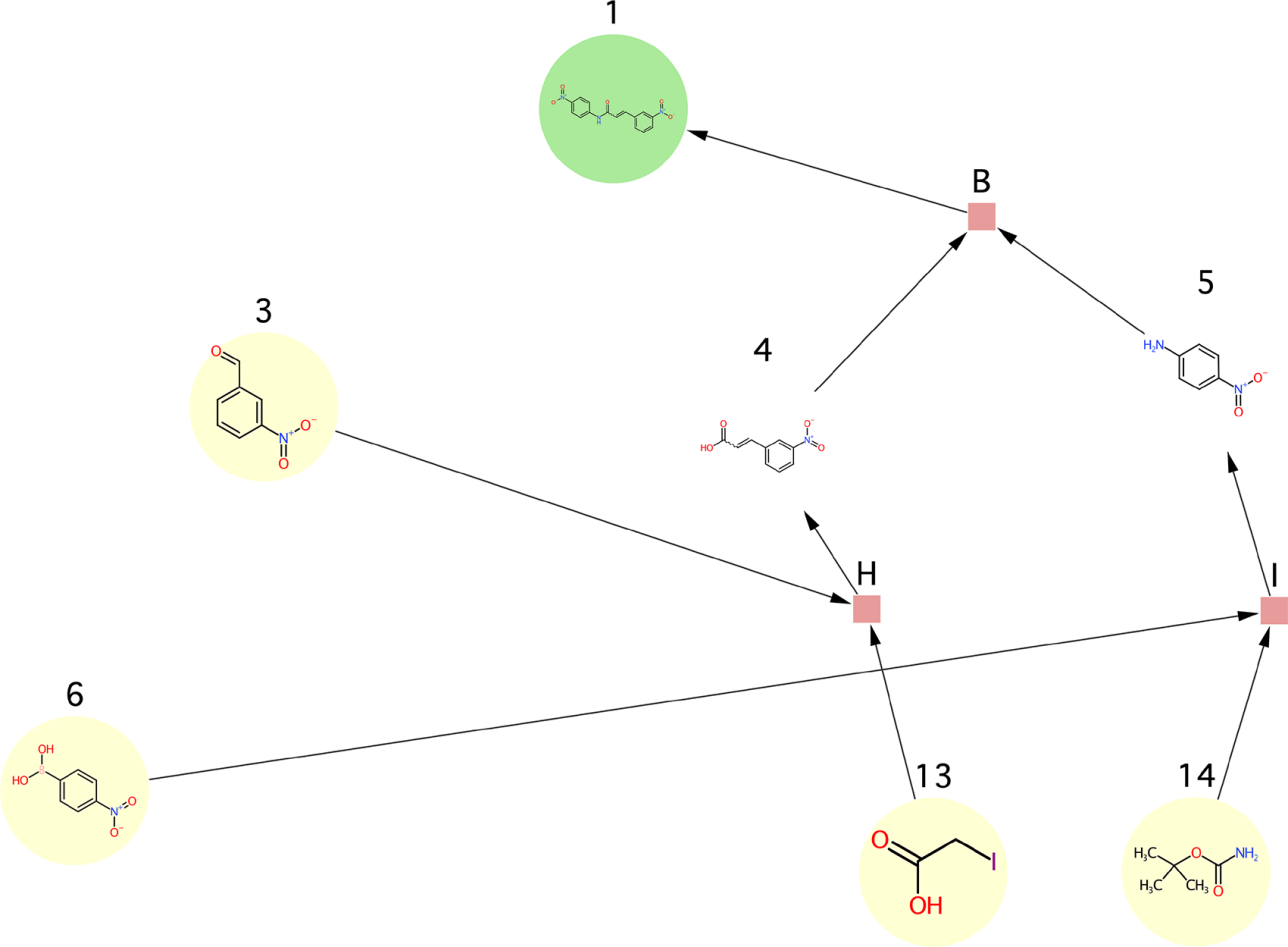
Use case 4 - synthesis graph pruned by the SGP algorithm.

In this last use case, we demonstrate that multiple substances can also be defined as “undesirable”. In an imaginary scenario, we assume all chlorine and bromine containing substances as undesirable. Considering the input graph shown in Figure 11A, this assumption makes 2, 7, 8, 9, 10, and 12 undesirable substances. The SGP algorithm correctly identifies that, given the input graph and the constraints, only one viable synthesis route remains (see Figure 15).

With the help of the above use cases, we demonstrated how the SGP algorithm correctly identifies viable synthesis routes (if exists) given an input synthesis graph and a set of constraints pertaining to the availability of substances from the inventory and whether or not they are allowed to be used in the synthesis. It is important to point out that the SGP algorithm does not try to identify or predict which substances should be considered desirable and if they are available from the inventory. As we discussed, these constraints need to be defined by the investigator and need to be provided to the SGP as input along with the underlying synthesis graph. Therefore, each time the SGP algorithm is run, the set of undesirable and inventory substances needs to be defined, that is, provided as input. This gives the flexibility of the SGP algorithm to run each analysis as an independent analysis. On the other hand, we imagine organizations can provide a default list of undesirable substances, e.g., controlled substances, which can be overridden by the investigator if need be. Naturally, the list of inventory substances is expected to be a dynamically changing list.

It should be noted, however, that there can be scenarios where a substance might be considered as undesirable only in the context of certain reaction types. The SGP algorithm currently cannot distinguish between reaction types. Therefore, once a substance is defined as undesirable in the input maybe due to reactivity reasons, for example, explosivity in a given reaction type, the SGP algorithm will make a stringent decision to eliminate the same substance from everywhere in the synthesis graph. However, it might be the case that the same substance is safe to use in the context of another reaction type; therefore, its elimination would not be necessary during the pruning process. We imagine in a future derivative version the SGP algorithm will be able to make such distinctions.

## Conclusions

In this study, we present the Synthesis Graph Pruning (SGP) algorithm and the corresponding mathematical framework, which provides an analytical solution for pruning a synthesis graph in the light of a set of undesirable substances. The SGP algorithm considers every substance as a starting material if they are readily available from the inventory. Therefore, even substances that represent an intermediate position or role in a synthesis graph are considered starting materials, if they are readily available. This distinction is important when the SGP algorithm identifies the viable synthesis routes given a set of undesirable substances.

The SGP algorithm represents an essential component in automated synthesis route planning, considering that an automated platform will be tasked to first identify all possible synthesis routes to a target molecule and then eliminate any routes that cannot be executed either due to the lack of any starting materials in the inventory or due to the harmful, toxic, or other adverse nature of starting materials and/or intermediates. The elimination of those synthesis routes from all possible synthesis routes results in the viable synthesis routes, as has been demonstrated via several use cases involving specific reactions extracted from a reaction knowledgebase.

## Data and Software Availability

The Python implementation of the SGP algorithm is available as open source code at the https://github.com/ncats/SGP repository.^39^ All synthesis graphs and additional input files as well as all output files are available for reproduction purposes at the same repository. Synthesis graphs are provided in GraphML (.graphml),^40^ XGMML (.xgmml),^41^ or Cytoscape session (.cys)^26^ format. The README.md file of the repository provides information on the location of these files.

## Supporting Information Available

Mathematical framework of the SGP algorithm, supplementary figures, input and output graphs in Cytoscape session, and custom style files (PDF)
