# Supplementary material for: Algorithm for the Pruning of Synthesis Graphs
Source: J Chem Inf Model. 2022 Apr 19;62(9):2226–38. doi: 10.1021/acs.jcim.1c01202 (PMC9093600; doi:10.1021/acs.jcim.1c01202)
Supplement: Supplementary file 1 — ci1c01202_si_001.pdf [file ci1c01202_si_001.pdf]

Supporting Information

Algorithm for the Pruning of Synthesis Graphs

Gergely Zahoránszky-Kőhalmi [1,*], Nikita Lysov[1], Ilia Vorontcov[1], Jeffrey Wang[1],

Jeyaraman Soundararajan[1], Dimitrios Metaxotos[1], Biju Mathew[1], Rafat Sarosh[1],

Samuel G. Michael[1], Alexander G. Godfrey[1]

[1]National Center for Advancing Translational Sciences, Rockville, Maryland 20850, United States

[*]Corresponding author:

Gergely Zahoránszky-Kőhalmi, PhD: gergely.zahoranszky-kohalmi@nih.gov

Mathematical Framework of the SGP Algorithm

Terminology

A *starting material* is a substance is readily available from the inventory or stock, i.e. "off the shelf". Naturally, a synthesis can only start from starting materials.

An *intermediate* in a multi-step synthesis context is a substance that is the product of a reaction and is also the reactant (or reagent) of a subsequent reaction. Note, that in synthesis planning, a substance that plays an intermediate role in the synthesis might be available from the inventory. Those substances will be considered as starting materials, despite their role in the synthesis.

A *target molecule* is a substance that one aims to synthesize, typically via a series of reactions.

A synthesis graph [1,2] G is a directed bipartite graph [3] consisting of set of reaction nodes R and a set of substance nodes S. The set of directed edges E is defined by the set of $(S \times R) \cup (R \times S)$ defined by 2-tuples in the form of $(s \in S, r \in R)$ or $(r \in R, s \in S)$. Each substance node $s \in S$ has an attribute, called type, $s.type \in \{starting\ material, intermediate, target\ molecule\}$. In G there can be only one node s whose *type* attribute is "*target molecule*", i.e. $|\{s_t \in S \mid s_t.type = target\ molecule\}| = 1$. Each edge $e \in E$ is associated with an edge attribute, called *role*, $e.role \in \{reactant, reagent, product\}$, so that $e.role \in \{reactant, reagent\} \mid e \in (S \times R)$ and $e.role = product \mid e \in (R \times S)$. In the current study, it is assumed that each reaction is irreversible, and has only one product. Furthermore, only one edge can exist between a given pair of s and r nodes.

Definitions

Def 1: A node n_1 is the *parent node* of n_2 if $(n_1 \in S, n_2 \in R) \in E$ or $(n_1 \in R, n_2 \in S) \in E$.

Def 2: A node n_2 is the *child node* of n_1 if $(n_1 \in S, n_2 \in R) \in E$ or $(n_1 \in R, n_2 \in S) \in E$.

Def 3: The number of parent nodes of a node n is defined by the function in-degree [4], i.e. $\deg_{in}(n)$.

Def 4: The number of child nodes of a node n is defined by the function out-degree [4], i.e. $\deg_{out}(n)$.

Def 5: A synthesis route W is directed acyclic subgraph of G comprised of substance and reaction nodes, starting from one or more starting material(s) $s_m \in S \mid \forall s_m : s_m.type = starting\ material$ and ending in $s_t \in S$ target molecule. It is true, that for every substance in W which are the products of a reaction in W, there exists a set of starting materials in W which are the starting nodes of paths leading to those products. One synthesis route is uniquely distinguished from any other synthesis routes in G by the set of its $\{s \in S\}$ substance and $\{r \in R\}$ reaction nodes, and by the sequence of nodes in all paths starting from the starting materials in W and ending in s_t.

Def 6: Given a synthesis graph G it is possible to define a set of *undesirable* substances and respective substance nodes, due to their undesirable properties or unavailability. The set of these nodes represents an input, i.e.: nodes to be eliminated G.

Def 7: A synthesis route is defined as *viable* if a path exists to any of its reaction nodes that i) starts from a starting material, and ii) it does not intersect any reaction nodes that is connected to an undesirable substance (see: *Def 6*). Synthesis routes that are note viable are *non-viable*.

Def 8: Let a reaction node $r_A \in R$ represent reaction "A" in a synthesis graph G. Reaction "A" becomes *undefined* if any of the substance nodes (reactant, reagent or product) connected to r_A gets removed from G. Consequently, r_A needs to be eliminated from G.

Observation 1: A synthesis graph G cannot be assumed to be a directed acyclic graph (DAG) [Ref:] as it may or may not contain cycles. In any case, it is possible to identify synthesis routes that are directed acyclic subgraphs of G.

Observation 2: The target molecule is represented by one particular node $s_t \in S$. s_t is distinguished from other nodes of G by the properties, that there exists a path from every $n \in \{S \cup R\} \setminus s_t$ to s_t and that $\deg_{\mathrm{out}}(s_t) = 0$.

Observation 3: The target molecule $s_t \in S$ only has one or more parent node(s).

Observation 4: For all parent node p of $s_t \in S$ it is true that $p \in R$.

Observation 5: If a substance node $s \in S$ has multiple child nodes, then it plays a role as a reactant or a reagent in multiple reactions $r_1, r_2, .. r_x \in R \mid x = \deg_{out}(s)$. The role of s can be different (reactant or reagent) in the context of its child nodes.

Observation 6: If a substance node $s \in S$ has multiple parent nodes, then it plays a role as a product in multiple reactions $r_1, r_2, .. r_x \in R \mid x = \deg_{in}(s)$.

Observation 7: *In the light of Observation 1*, one needs to consider the following scenario. Let's assume there is an $s_m \in S, s_m.type = starting\ material$ substance node involved as a starting material in a synthesis route W involving reaction nodes $r_1, r_2, .. r_i$. Let's assume, that there is another synthesis route Y of which r_1', r_2' reaction nodes are constituents of, and which also involves s_m so that it is a product of r_2'. Therefore, it is true that $\deg_{in}(s_m) \geq 1$. This is a valid synthesis scenario, that is, one can use s_m as readily available starting material in W, however, there might be another reaction route Y that involves s_m as a product, i.e.: s_m has a reaction parent node. See: *FigS1*.

Theorem 1: Upon the removal of a reaction node $r \in R$ from G, any parent nodes (substances) $p \in S$ of r will need to be eliminated from G if, and only if $\deg_{out}(p) = 0$ after the removal of r. This also holds true, if multiple reaction nodes are eliminated from G before assessing the $\deg_{out}(p) = 0$ condition for each implicated substance nodes.

Proof: Let's consider a substance node $s_p \in S$ and a reaction node $r_e \in R$ so that an edge exists between them where s_p acts as the parent node, i.e.: $(s_p, r_e) \in E$. When removing r_e from G, only two cases can hold true.

In the first case, $\deg_{out}(s_p) = 0$, that is, no reaction node exists any longer in G whose parent node (either as reactant or reagent) would be s_p. At this point, it is, however, possible that s_p participates in one or more edges $e \in E$, where it acts as a child node. From a synthesis route point of view, the only relevant question to ask at this point is: is there any route involving s_p that leads to the target molecule s_t? It is easy to see that this is not the case, since the only reaction node (r_e) whose parent node was s_p, at this point was already eliminated from G.

In the second case, i.e.: $\deg_{out}(s_p) \geq 1$, regardless of how many parent nodes s_p might have, it cannot be eliminated from G as it has at least one child node $r \in \{R \setminus r_e\}$, that is on a route that still has a chance to lead to s_t. That is, s_p is either a reactant or reagent of at least one reaction that is the constituent of an alternative synthesis route to s_t. ■

Theorem 2: Upon the removal of a reaction node $r \in R$ from G, any child nodes (substances) $c \in S$ of r will need to be eliminated from G if, and only if $\deg_{in}(c) = 0 \mid c.type \neq starting\ material$ after the removal of r. This also holds true, if multiple reaction nodes are eliminated from G before assessing the $\deg_{in}(c) = 0 \mid c.type \neq starting\ material$ condition for each implicated substance nodes.

Proof: This proof is derived in an analogous manner to Proof 1. Let's consider a substance node $s_c \in S \mid s_c.type \neq starting\ material$ and a reaction node $r_e \in R$ so that an edge exists between them where s_c acts as the child node, i.e.: $(r_e, s_c) \in E$. When removing r_e from G, only two cases can hold true. In the first case, $\deg_{in}(s_c) = 0$, that is, no reaction node exists any longer in G whose child node (as product) would be s_c. At this point, it is, however, possible that s_c participates in one or more edges $e \in E$, where it acts as a parent node. From a synthesis route point of view, the only relevant question to ask at this point is: is there any route involving s_c that leads to the target molecule s_t? It is easy to see that this is not the case, since the only reaction node (r_e) whose child node (product) was s_c, at this point was already eliminated from G. This means that s_c in this scenario "cannot be synthesized". Thus, any synthesis attempt starting from this substance is undesirable.

However, if it were true that $s_c.type = starting\ material$ then it was not required that it is connected to a reaction as a product, so it would not be eliminated from G even if the condition $\deg_{in}(s_c) = 0$ held true at any point (see: *Observation 7).*

In the second case, i.e.: $\deg_{in}(s_c) \geq 1$, regardless of how many child nodes s_c might have, it cannot be eliminated from G as it has at least one parent node $r \in \{R \setminus r_e\}$, that provides an alternative synthesis route to s_c that does not involve the same (or other) undesirable intermedier that led to the elimination of r_e in the first place. ■

Observation 8: In a scenario, when a substance node $s_c \in S$ has at least two parent reaction nodes and all of these reaction nodes are eliminated from the synthesis graph, then the assessment of the criterion whether s_c should be eliminated from G (see: *Theorem 1*) is independent of the sequence of removal of the parent reaction nodes. Furthermore, the assessment of the criterion can happen after the simultaneous removal of more than one, or all of the parent reaction nodes. In an analogous manner, the same observation can be made for assessing the removal criteria (see*: Theorem 2*) for a substance node $s_p \in S$ if it has at least two child reaction nodes.

Def 9: A local rule set to prune a synthesis graph G can be established based on the above definitions, observations and *Theorem 1* and *Theorem 2*:

- Substances flagged as undesirable are removed from G.
- Reaction nodes are removed if they were immediate neighbors of a substance node before its deletion from G, as the implicated reaction became undefined (see: *Def 8*).

- A substance node $s \in S$ is deleted from G if the $\deg_{out}(s) = 0$ elimination criterion becomes true upon the deletion of one or more of its child reaction node(s) $\{r \in R\}$ (see: *Theorem 1*).
- A substance node $s \in S$ is deleted from G if the $\deg_{in}(s) = 0$ elimination criterion becomes true upon the deletion of one or more of its parent reaction node(s) $\{r \in R\}$, unless s is a starting material (see: *Theorem 2*).

Theorem 3: Let's consider a synthesis graph G, and one of its viable synthesis routes W (see: *Def 7*), that involves a succession of reaction nodes $r_1,\ r_2,\ r_3,\ ..,\ r_i$, where r_1 is connected to one or more $\{s_m \mid \forall s_m: s_m.type = starting\ material\ \}$ starting materials, and r_i is the parent node of target molecule s_t. Let's assume that two reaction nodes r_2 an r_3 are separated by a substance node $s \neq s_t$, so that r_2 is the parent node of s, whereas $r_3$is the child node of s. Furthermore, s is connected to a reaction node $r' \notin W$ that is not part of the synthesis route W. In this case, it holds true, that the elimination of r' from G will leave the viable synthesis route W intact. That is, all reaction nodes in W and their associated substance nodes will not be removed form G.

Proof:

In the relation of r_2, r_3, s and r' four distinct cases exist. Since no other cases are possible, conclusions drawn from these cases generalize to any G. Here, we consider the four possible cases.

Case 1: $s \mid s.type \neq starting\ material$ is the child node of r_2 and the parent node of r_3 and r' is the parent node of s, see: *Fig S2*.

Once r' is eliminated from G, we need to assess whether s, a child node of r' needs to be eliminated as well. According to *Theorem 2*, s needed to be eliminated if the condition $\deg_{\text{in}}(s) = 0 \mid s.type \neq starting\ material$ held true. Considering that s is a child node of r_2, which is not affected by the deletion of r', the above condition does not hold true. Therefore, s does not need to be eliminated from G, leaving W intact.

Case 2: $s \mid s.type \neq starting\ material$ is the child node of r_2 and the parent node of r_3 and r' is the child node of s, see: *Fig S3.*

Once r' is eliminated from G, we need to assess whether s, a parent node of r' needs to be eliminated as well. According to *Theorem 1*, if $\deg_{\text{out}}(s) = 0$ holds true, then s would need to be eliminated. Considering that s is a parent node of r_3, which is not affected by the deletion of r', the above condition does not hold. Therefore, s does not need to be eliminated, leaving W intact.

Case 3: $s \mid s.type = starting\ material$ is the parent node of r_2, and r' is the parent node of s, see: *Fig S4*.

Since s is the child node of r', upon the removal of r', we need to assess, if the elimination criterion according to *Theorem 2* holds true for s. The removal of r' will lead to the condition of $\deg_{in}(s) = 0$ being true, however, s is a starting material. Therefore, overall elimination criterion does not hold true. Consequently, s does not need to be removed from G, leaving W intact.

Case 4: $s \mid s.type = starting\ material$ is the parent node of r_2 and r' is the child node of s, see: *Fig S5*.

It can be seen, that in an analogous manner to case 2, s does not need to be eliminated from G, as $\deg_{\mathrm{out}}(s) = 0$ will not hold true despite the deletion r', leaving W intact.

We could consider two additional, only hypothetical cases. However, in the light of the conditions set forth in this theorem, we show that these two cases lead to contradiction.

Hypothetical case 1: $s \mid s.type \neq starting\ material$ is a parent node of r_2, and r' is a parent node of s, and s has no other neighbors. See: *Fig S6*.

The elimination of r' would lead to this condition being true: $\deg_{in}(s) = 0$. Considering that s is not a starting material, the elimination condition for s according to *Theorem 2* would hold

true. However, this leads to a contradiction. Namely, W is not a viable synthesis route (see: *Def 7*), and r' would be actually a constituent of W.

Hypothetical case 2: $s \mid s.type \neq starting\ material$ is a parent node of r_2, and r' is a parent node of s, and there exist another reaction node r_x that is the parent node of s, but r_x is not part of W. Furthermore, s has no other neighbors. See: *Fig S7*.

Upon removal of r' the elimination criterion, according to *Theorem 2*, for s would not hold true, as it has an additional parent node, r_x. Therefore, after the elimination of r', $\deg_{in}(s) = 1$, rendering the elimination criterion false. However, this scenario would mean that r_x must be the part of W, which leads to a contradiction. Namely, according to the hypothetical case 2, r_x is not a constituent of W.

Taking all the above cases into consideration, we conclude, that the elimination of a reaction node r' will leave a viable synthesis route W intact, as long as r' is not a constituent of W. ■

Theorem 4: Given a valid synthesis route W in a synthesis graph G, if two of the constituent substance nodes (s_1 and s_2) of W are connected by a subgraph Y that is not part of W, then the deletion of that subgraph will leave W intact.

Proof: The fact that s_1 and s_2 are connected by Y implies that there exists a sequence of nodes that constitutes a path P between s_1 and s_2, if we ignore the directed nature of G, and assume

that all edges are undirected. For clarification s_1 and s_2 are not part of P. However, the terminal nodes of P are reaction nodes, since G is a bipartite graph. As it was shown in the proof of *Theorem 3*, in the relation of a substance node $s \in W$ and a reaction node $r' \notin W$ the removal of r' requires the assessment of the properties of only the substance node s in order to decide whether s would need to be eliminated as well. Therefore, deletion of P reduces to assessing the elimination criteria for both s_1 and s_2 independently, according to *Theorem 1* and *Theorem 2*. Of course, for the assessment of the criteria, we need to consider the original directed edges between s_1, s_2 and the two terminal reaction nodes. Therefore, it can be seen in an analogous manner as in the proof of *Theorem 3*, that W is left intact upon the deletion of the subgraph Y which P was the constituent of. ■

Theorem 5: The local rule set outlined in *Def 9* is alone sufficient to serve as the decision-making mechanism of an algorithm that will prune a synthesis graph G with a target molecule $s_t, s_t.type = target\ molecule$ in a specific manner: taking a set of substance nodes $I = \{\forall s_i \in S \mid I \neq S,\ s_i \neq s_t\}$ and G as input, i) it will completely remove all synthesis routes involving a node $n \in I$ starting from the starting materials of the synthesis routes, and ii) it will not affect *viable* synthesis routes (see: *Def 7*) which represent synthesis alternatives, i.e. they don't involve any of the undesirable substances defined by the input set.

Proof: Let a synthesis graph G consist of a set of substances nodes S and of a set of reaction nodes R. The set of input nodes $I = \{\forall s_i \in S\}$ consist only of substance nodes. The elimination of these nodes gives rise to a set of reaction nodes $R_1 = \{\forall r_1 \in R\}$ which, in turn, also needs to

be eliminated from G, as all reaction of R_1 has become undefined (see: *Def 8*). In consequence of the removal of the reaction nodes in R_1, a set of substance nodes $S_1 = \{\forall s_1 \in S\}$ emerges. Nodes in S_1 were connected to at least one of reactions nodes in R_1 before their removal. Therefore, we need to assess the removal criteria for each $s_1 \in S_1$ as described by the local rule set (see: *Def 9*). In the light of *Observation 8*, all nodes in R_1 can be eliminated from G before assessing the elimination criteria according to the local rule set for nodes in S_1.

Let $S_{1e} \subseteq S_1$ denote a subset of nodes of S_1, that end up being eliminated from G based on the local rule set. Unless G has become empty at this point, then another set of reaction nodes ($R_2 = \{\forall r_2 \in \{R \setminus R_1\}\}$ emerges, that have become undefined upon the removal of S_{1e}, provided that $S_{1e} \neq \emptyset$. Elimination of the reaction nodes in R_2 give rise to a set of substance nodes $S_2 = \{\forall s_2 \in \{S \setminus S_{1e}\}\}$ which are also candidates for elimination in the light of the local rule set. Repeating this process gives rise to pairs of $(R_x, S_x) \mid x \in \mathbb{N}$, until no substance node in S_x is eliminated or G becomes empty. So far, we proved that the process of iteratively removing nodes from G based on the input set I and the local rule set will terminate in a deterministic manner. We also proved in *Theorem 3* and *Theorem 4* that the elimination of the input substance nodes, and subsequently all the other reaction nodes from a set R_x, and any substance nodes in the corresponding set S_x will leave all viable synthesis routes intact. Consequently, the elimination process will only remove substance and reaction nodes that are not involved in any viable synthesis routes. Therefore, all non-viable synthesis routes will be eliminated completely once the iterative elimination process terminates. ■

Pseudo Code of the SGP Algorithm

Algorithm SGP

Input: synthesis graph *G*
Input: set of undesirable substances *lo_substances*

```
function flagNodes (neighbors = [], target_list = [])

    for all neighbor in neighbors  do
        if  neighbor not in target_list then
            target_list.add (neighbor)
        end if
    end for

return (target_list)


function markNodeForDeletion (node, nodes_2b_deleted = [])

    if node not in nodes_2b_deleted  then
        nodes_2b_deleted.add(node)
    end if

return (nodes_2b_deleted)


function SGP (G, lo_substances = [])

    Variable: list of nodes n_idel
    Variable: list of nodes n_sdel_p
    Variable: list of nodes n_sdel_c
    Variable: list of nodes deleted_nodes
    Variable: node n_i
    Variable: node n_s


    n_idel.merge (lo_substances)

    while (not n_idel.empty() or not n_sdel_p.empty() or not n_sdel_c.empty()) do
```

```
while not n_idel.empty() do

    n_i = n_idel.pop()

    if n_i in G.nodes then

        parent_neighbors = get_parent_nodes (G, n_i)
        child_neighbors = get_child_nodes (G, n_i)


        if n_i.attribute('node_type') == 'Substance' then

            n_idel = flagNodes (parent_neighbors, n_idel)
            n_idel = flagNodes (child_neighbors, n_idel)

        else

            n_sdel_p = flagNodes (parent_neighbors, n_sdel_p)
            n_sdel_c = flagNodes (child_neighbors, n_sdel_c)

        end if

        G.remove_node(n_i)


    end if

end while


while not n_sdel_p.empty() do
    n_s = n_sdel_p.pop()

    if get_out_degree(G, n_s) == 0:
        n_idel = markNodeForDeletion (n_s, n_idel)
end while

while not n_sdel_c.empty() do
    n_s = n_sdel_c.pop()

    if (get_in_degree(G, n_s) == 0 and n_s.attribute('srole') != 'SM') then
        n_idel = markNodeForDeletion (n_s, n_idel)
```

end if

end while

end while

return (*G*)

G = **SGP** (*G*, *lo_substances*)

Time Complexity Analysis of the SGP Algorithm

Here, we present the computational time-complexity of the SGP algorithm considering worst case scenarios.

Let's consider a synthesis graph G consisting of S substance, and R reaction nodes, i.e.: G has $Z = S + R$ nodes in total. Furthermore, let I denote the input set of undesirable substances. The adjacency information of nodes are stored in an *adjacency list* format [5]. That is, each node of G is associated with two lists which contain the child and parent nodes of a particular node, respectively. Also, the number of child and parent nodes, i.e.: the size of the respective adjacency lists, of each node is maintained as node attributes.

At the initial phase (Step 1 in *Fig 1*) of the SGP algorithm all the neighbors of the substances in the input set I need to be eliminated. Although unrealistic, all substances of G could be included in I. Therefore, the identification of reaction nodes to be marked for deletion might require a maximum of $S \times R$ steps. This can be approximated by Z^2 steps.

In Step 2, a maximal of S are eliminated, which requires updating the adjacency lists of each reaction nodes. This requires a maximum of $R \times S$ steps, which can be estimated as Z^2. Also, we need to remove $2 \times S$ adjacency lists associates with the deleted substance nodes, which can be approximated by $2Z$ steps.

Next, the neighbors of each reaction nodes marked for deletion are identified in Step 3. This can be approximated with Z^2 steps.

In step 4, the elimination of the marked reaction nodes requires $Z^2 + 2Z$ steps in order to update and remove the respective adjacency lists. The inspection of any substance nodes potentially marked for inspection requires $2S$ steps, as the in-degree and out-degree [4] properties are node attributes that can be looked-up. This process can be approximated by $2Z$ steps.

It can be seen that computational time requirements after the Step 4 can be estimated by repeating the above analysis at most $(Z-2)/2$ times, considering that in each iteration at least one reaction and one substance node need to be removed for the iteration to continue. Therefore, the computational complexity of the SGP algorithm can be estimated according to *Eq 1*.

$$O = Z^2 + \frac{Z}{2}(3Z^2 + 6Z) = 4Z^2 + \frac{3}{2}Z^3 \approx Z^3 \qquad (1)$$

Therefore, the computational time complexity of the SGP algorithm is bounded by the cubic function of the total number of nodes in the synthesis graph at hand. A tighter bound of this for the computational complexity is likely to be found, however, the more in-depth analysis is outside of the scope of this study. Furthermore, it might be possible to find points of optimization of the SGP algorithm which might result in more efficient computational time complexity. However, given the current state-of-the-art, typical synthesis graphs are anticipated to be comprised of nodes in the magnitude of thousands. Therefore, the SGP algorithm is expected to process a typical synthesis graph in reasonable runtime [6].

Figures

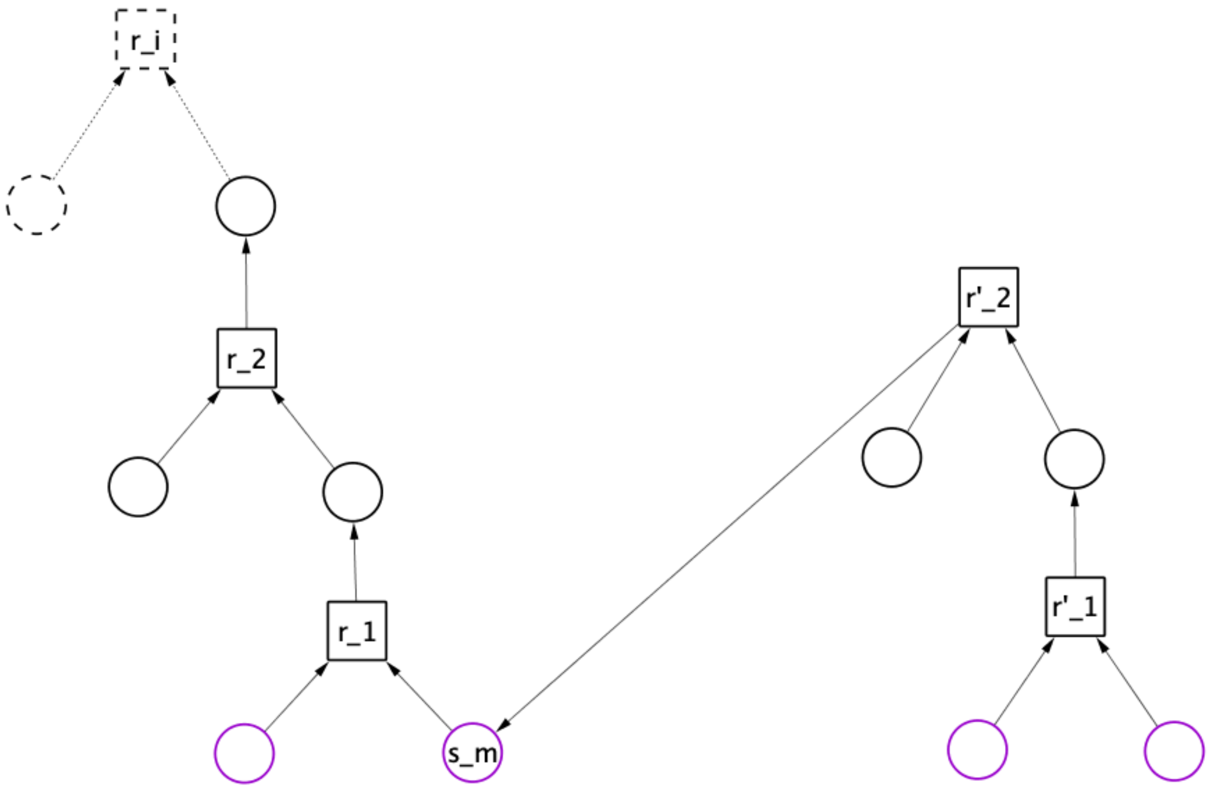

Fig S1. **Observation 7.** Reaction nodes are represented by squares, whereas substance nodes by circles. Nodes and edges drawn with dotted lines indicate that such nodes and edges may or may not exist. Nodes with purple outline indicate starting materials.

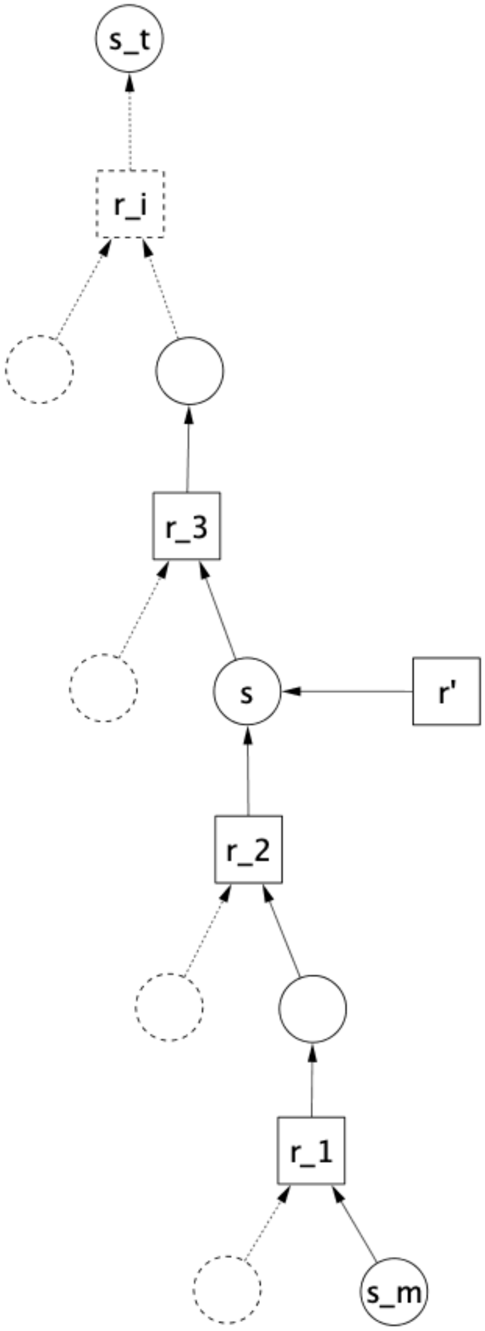

Fig S2. **Theorem 3, case 1.** Reaction nodes are represented by squares, whereas substance nodes by circles. Nodes and edges drawn with dotted lines indicate that such nodes and edges may or may not exist in the synthesis route W.

W.

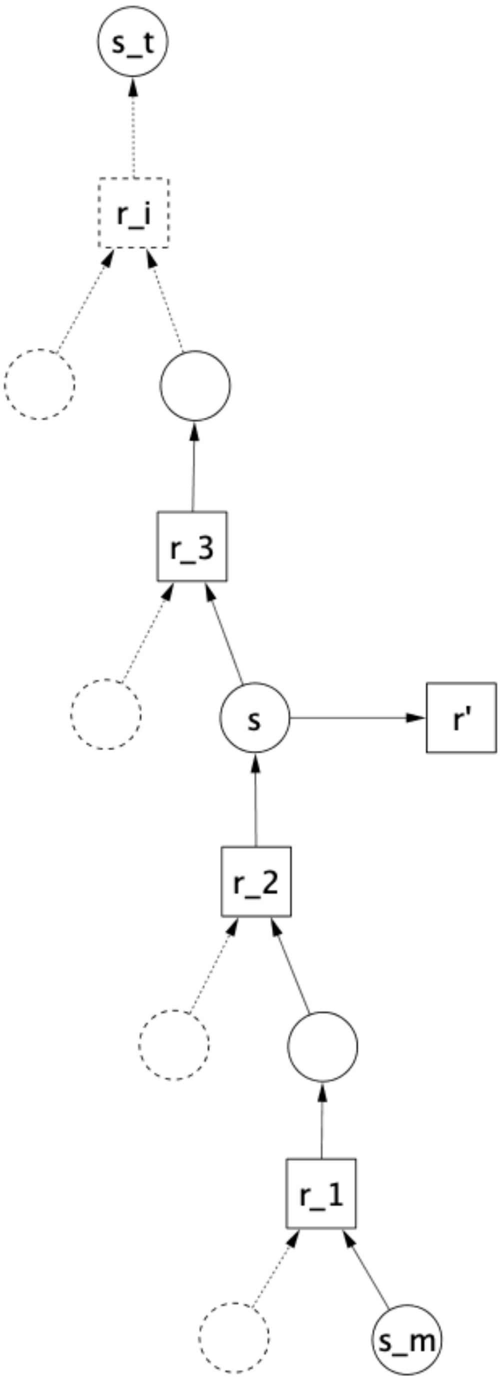

Fig S3. **Theorem 3, case 2.** Reaction nodes are represented by squares, whereas substance nodes by circles. Nodes and edges drawn with dotted lines indicate that such nodes and edges may or may not exist in the synthesis route W.

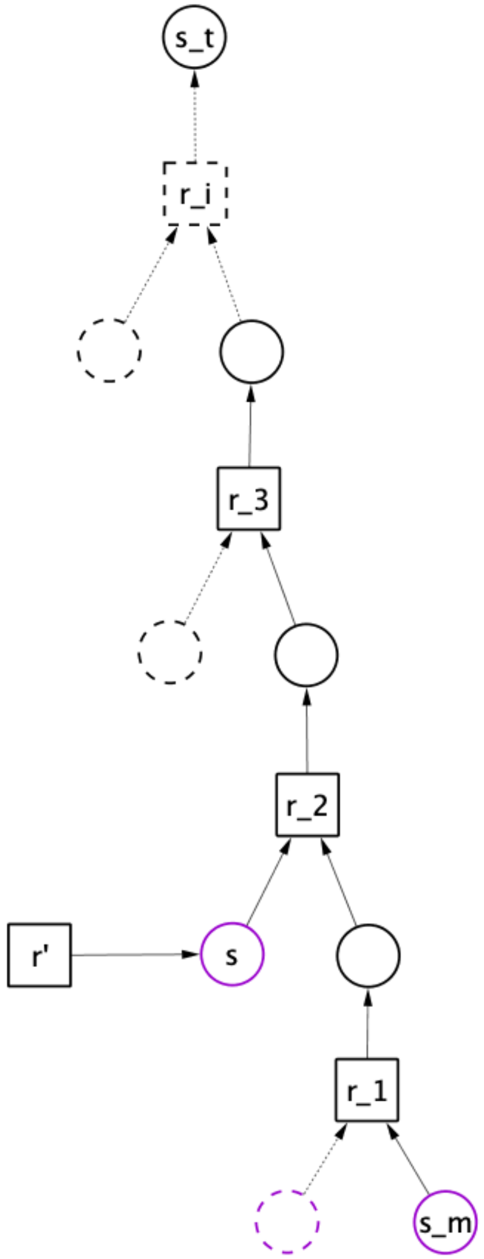

Fig S4. **Theorem 3, case 3.** Reaction nodes are represented by squares, whereas substance nodes by circles. Nodes and edges drawn with dotted lines indicate that such nodes and edges may or may not exist in the synthesis route W. Nodes representing starting materials are highlighted by purple.

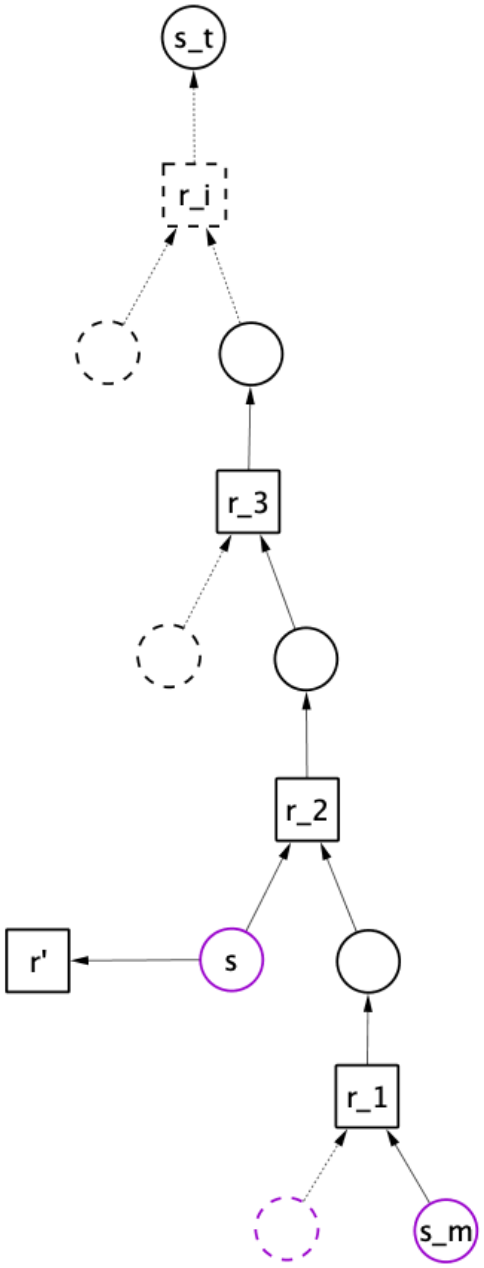

Fig S5. **Theorem 3, case 4.** Reaction nodes are represented by squares, whereas substance nodes by circles. Nodes and edges drawn with dotted lines indicate that such nodes and edges may or may not exist in the synthesis route W. Nodes representing starting materials are highlighted by purple.

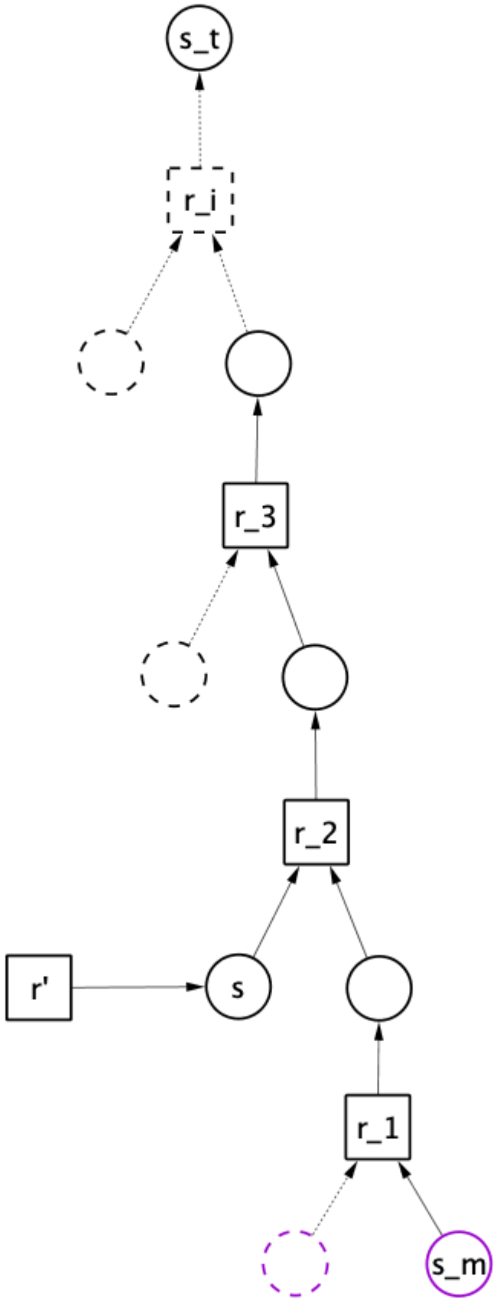

Fig S6. **Theorem 3, hypothetical case 1.** Reaction nodes are represented by squares, whereas substance nodes by circles. Nodes and edges drawn with dotted lines indicate that such nodes and edges may or may not exist in the synthesis route W. Nodes representing starting materials are highlighted by purple.

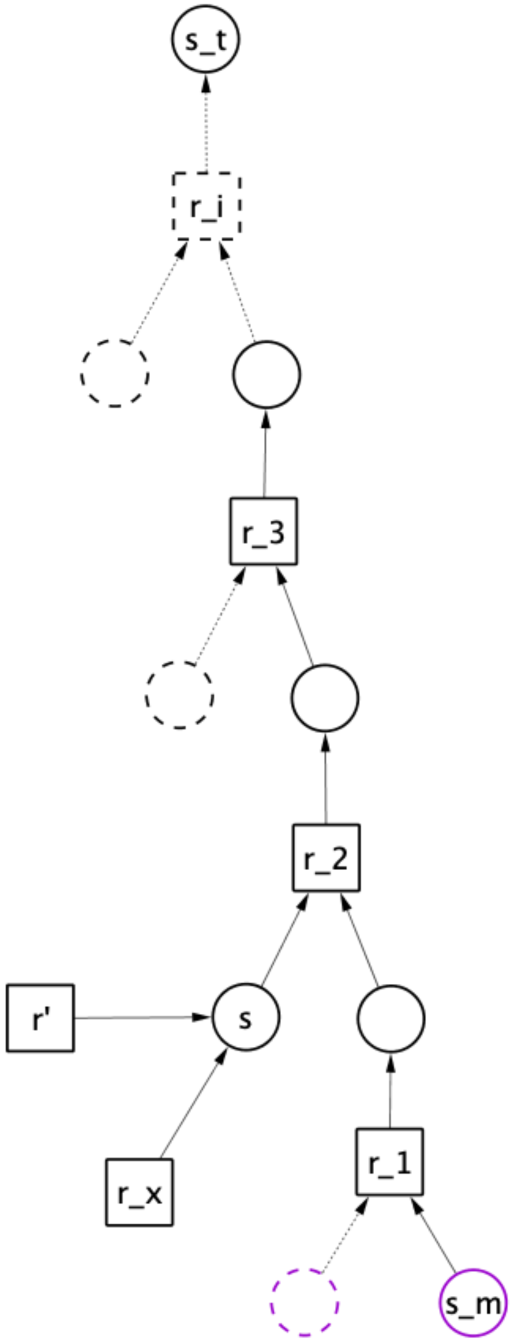

Fig S7. **Theorem 3, hypothetical case 2.** Reaction nodes are represented by squares, whereas substance nodes by circles. Nodes and edges drawn with dotted lines indicate that such nodes and edges may or may not exist in the synthesis route W. Nodes representing starting materials are highlighted by purple.

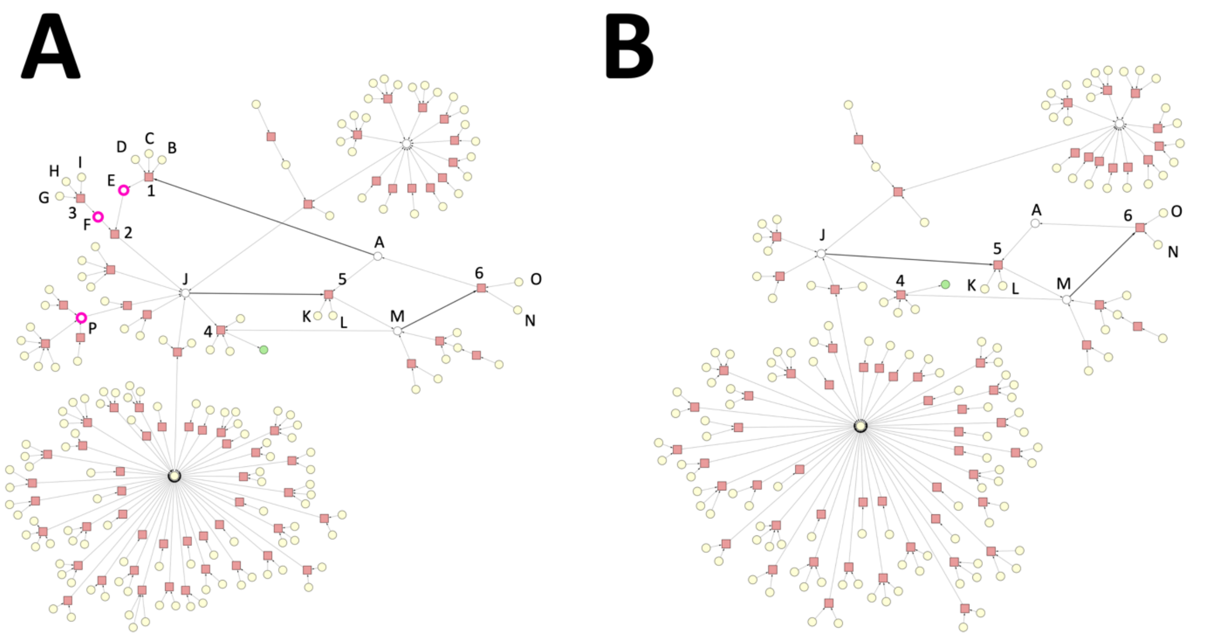

Fig S8. **Case 8.** Reaction nodes are represented by squares, whereas substance nodes by circles. Color code of substance nodes; green: target molecule, yellow: starting material, magenta outline: undesirable substance, white: intermediate. Note, the labels of nodes are preserved across Case 6, 7 and 8, only an additional substance was given the label “P” in Case 8 as compared to Case 6 and 7. **A**: Original synthesis graph. Substances “E”, “F”, “P” are intermediates and were marked as undesirable substances. **B**: Pruned synthesis graph.

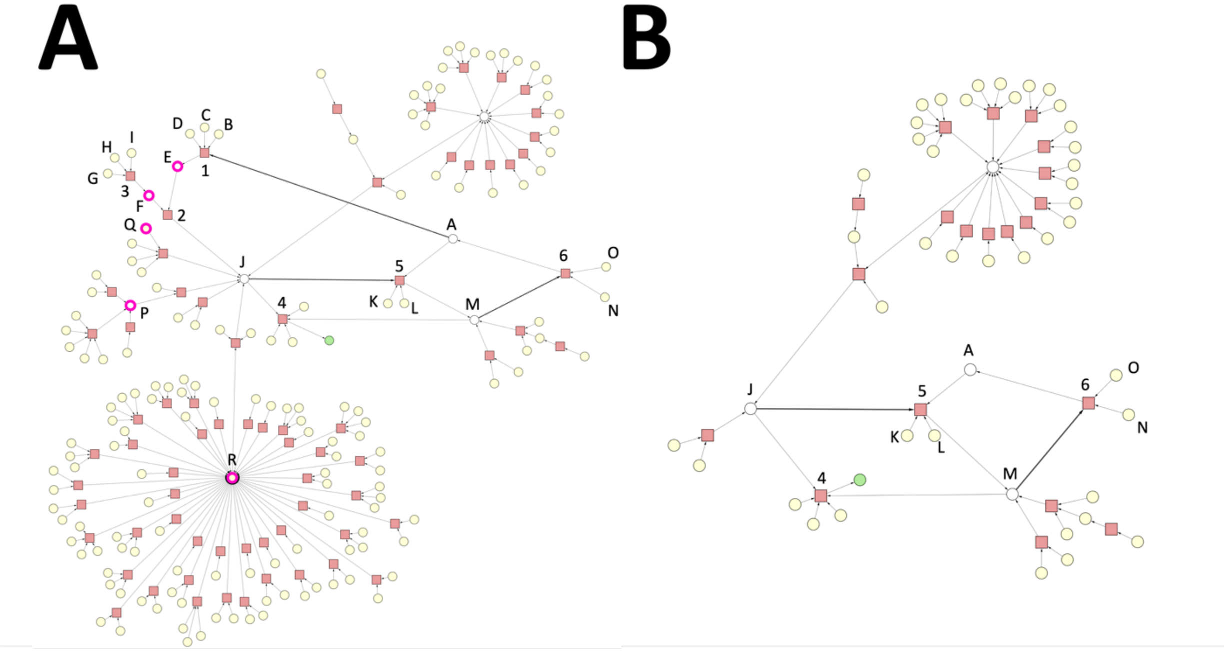

Fig S9. **Case 9.** Reaction nodes are represented by squares, whereas substance nodes by circles. Color code of substance nodes; green: target molecule, yellow: starting material, magenta outline: undesirable substance, white: intermediate. Note, the labels of nodes are preserved across Case 6, 7, 8 and 9, only two additional substance were given the label “Q” and “R” in Case 9 as compared to Case 8. **A**: Original synthesis graph. Substances “E”, “F”, “P” are intermediates and were marked as undesirable substances. Substances “Q” and “R” are starting materials and were also marked as undesirable substances. **B**: Pruned synthesis graph.

Tables

InChI-Key[7]	SMILES[8,9]	IUPAC Name[10,11]	ID
BMDNOIHVFOBSAS-UHFFFAOYSA-N	[O-][N+](c(cc1)ccc1NC(C=Cc1cccc([N+]([O-])=O)c1)=O)=O	3-(3-nitrophenyl)-N-(4-nitrophenyl)prop-2-enamide	1
AZURFBCEYQYATI-UHFFFAOYSA-N	O=C(CCl)Nc1ccc([N+](=O)[O-])cc1	2-chloro-N-(4-nitrophenyl)acetamide	2
ZETIVVHRRQLWFW-UHFFFAOYSA-N	O=Cc1cccc([N+](=O)[O-])c1	3-nitrobenzaldehyde	3
WWXMVRYHLZMQIG-UHFFFAOYSA-N	O=C(O)C=Cc1cccc([N+](=O)[O-])c1	3-(3-nitrophenyl)prop-2-enoic acid	4
TYMLOMAKGOJONV-UHFFFAOYSA-N	Nc1ccc([N+](=O)[O-])cc1	4-nitroaniline	5
NSFJAFZHYOAMHL-UHFFFAOYSA-N	O=[N+]([O-])c1ccc(B(O)O)cc1	(4-nitrophenyl)boronic acid	6
VXIVSQZSERGHQP-UHFFFAOYSA-N	NC(=O)CCl	2-chloroacetamide	7
BNWCETAHAJSBFG-UHFFFAOYSA-N	CC(C)(C)OC(=O)CBr	tert-butyl 2-bromoacetate	8
KDPAWGWELVVRCH-UHFFFAOYSA-N	O=C(O)CBr	2-bromoacetic acid	9
FOCAUTSVDIKZOP-UHFFFAOYSA-N	O=C(O)CCl	2-chloroacetic acid	10
ZNRGSYUVFVNSAW-UHFFFAOYSA-N	O=[N+]([O-])c1cccc(B(O)O)c1	(3-nitrophenyl)boronic acid	11
POAWTYXNXPEWCO-UHFFFAOYSA-N	O=C(O)C=CBr	3-bromoprop-2-enoic acid	12
JDNTWHVOXJZDSN-UHFFFAOYSA-N	O=C(O)CI	2-iodoacetic acid	13
LFKDJXLFVYVEFG-UHFFFAOYSA-N	CC(C)(C)OC(N)=O	tert-butyl carbamate	14

Table S1. Substances involved in use cases. Substance nodes in the graphs of the use cases are numbered in correspondence to the IDs shown in this table.

References

(1) Shibukawa, R.; Ishida, S.; Yoshizoe, K.; Wasa, K.; Takasu, K.; Okuno, Y.; Terayama, K.; Tsuda, K. CompRet: A Comprehensive Recommendation Framework for Chemical Synthesis Planning with Algorithmic Enumeration. *J. Cheminform.* **2020**, *12* (1), 52. https://doi.org/10.1186/s13321-020-00452-5.

(2) Bradshaw, J.; Paige, B.; Kusner, M.; Segler, M.; Hernández-Lobato, J. M. Barking up the Right Tree: An Approach to Search over Molecule Synthesis {DAGs}. In *Advances in Neural Information Processing Systems 33*; Curran Associates, Inc., 2020; pp 6852–6866.

(3) Pavlopoulos, G. A.; Kontou, P. I.; Pavlopoulou, A.; Bouyioukos, C.; Markou, E.; Bagos, P. G. Bipartite Graphs in Systems Biology and Medicine: A Survey of Methods and Applications. *Gigascience* **2018**, *7* (4). https://doi.org/10.1093/gigascience/giy014.

(4) Batool, K.; Niazi, M. A. Towards a Methodology for Validation of Centrality Measures in Complex Networks. *PLoS One* **2014**, *9* (4), e90283. https://doi.org/10.1371/journal.pone.0090283.

(5) Coimbra, M. E.; Francisco, A. P.; Veiga, L. An Analysis of the Graph Processing Landscape. *J. Big Data* **2021**, *8* (1), 55. https://doi.org/10.1186/s40537-021-00443-9.

(6) Genheden, S.; Engkvist, O.; Bjerrum, E. Clustering of Synthetic Routes Using Tree Edit Distance. *J. Chem. Inf. Model.* **2021**, *61* (8), 3899–3907. https://doi.org/10.1021/acs.jcim.1c00232.

(7) Heller, S.; McNaught, A.; Stein, S.; Tchekhovskoi, D.; Pletnev, I. InChI - the Worldwide Chemical Structure Identifier Standard. *J. Cheminform.* **2013**, *5* (1), 7.

https://doi.org/10.1186/1758-2946-5-7.

(8) Weininger, D. SMILES, a Chemical Language and Information System. 1. Introduction to Methodology and Encoding Rules. *J. Chem. Inf. Model.* **1988**, *28* (1), 31–36. https://doi.org/10.1021/ci00057a005.

(9) Weininger, D.; Weininger, A.; Weininger, J. L. SMILES. 2. Algorithm for Generation of Unique SMILES Notation. *J. Chem. Inf. Model.* **1989**, *29* (2), 97–101. https://doi.org/10.1021/ci00062a008.

(10) INTERNATIONAL UNION OF PURE AND APPLIED CHEMISTRY (IUPAC) - Nomenclature https://iupac.org/what-we-do/nomenclature/.

(11) ChemAxon Ltd., Marvin Suite. IUPAC Names of Molecules Were Generated with ChemAxon's MarvinSketch 16.12.12.
